# Training and implementation of handheld ultrasound technology at Georgetown Public Hospital Corporation in Guyana: a virtual learning cohort study

**DOI:** 10.3352/jeehp.2023.20.11

**Published:** 2023-04-04

**Authors:** Michelle Bui, Adrian Fernandez, Budheshwar Ramsukh, Onika Noel, Chris Prashad, David Bayne

**Affiliations:** 1 Department of Urology, University of California, San Francisco School of Medicine, San Francisco, CA, USA; 2 Urology Division of General Surgery, Georgetown Public Hospital Corporation, Georgetown, Guyana; 3 Department of Urology, University of Texas Health San Antonio, San Antonio, TX, USA; Hallym University, Korea

**Keywords:** Educational measurement, Global health, Online systems, Ultrasonography, Urology

## Abstract

A virtual point-of-care ultrasound (POCUS) education program was initiated to introduce handheld ultrasound technology to Georgetown Public Hospital Corporation in Guyana, a low-resource setting. We studied ultrasound competency and participant satisfaction in a cohort of 20 physicians-in-training through the urology clinic. The program consisted of a training phase, where they learned how to use the Butterfly iQ ultrasound, and a mentored implementation phase, where they applied their skills in the clinic. The assessment was through written exams and an objective structured clinical exam (OSCE). Fourteen students completed the program. The written exam scores were 3.36/5 in the training phase and 3.57/5 in the mentored implementation phase, and all students earned 100% on the OSCE. Students expressed satisfaction with the program. Our POCUS education program demonstrates the potential to teach clinical skills in low-resource settings and the value of virtual global health partnerships in advancing POCUS and minimally invasive diagnostics.

## Graphical abstract

[Fig f2-jeehp-20-11]

## Background/rationale

According to the 2016 RAD-AID Conference on international radiology for developing countries, over half of the world lacks access to radiology [1]. This vast health disparity remains despite expanded global outreach efforts to low and middle-income countries (LMICs) and technological advances in the past 15 years, which have rendered imaging more accessible and less costly [1]. Notably, point-of-care ultrasound (POCUS) education programs in LMICs have demonstrated success in training providers in resource-limited settings [2,3]. Georgetown Public Hospital Corporation (GPHC) is a major public surgical facility in Guyana, an LMIC, yet urologists often lack point of care imaging studies in urologic clinics to guide their clinical decision making. This can impede the provision of quality care to patients. For instance, urologists at GPHC diagnose urinary retention by catheterizing their patients for post-void residual bladder volumes instead of using an ultrasound scanner. GPHC exemplifies the dearth of POCUS training in LMICs in the field of urology, despite a growing interest in global health in the field [4].

## Objectives

With an understanding of the imaging circumstances at GPHC, we piloted a virtual POCUS education program using the Butterfly iQ—a smart phone compatible, handheld ultrasound—in the evaluation of urology patients. We hypothesized that trainees would meet previously established standards for performing POCUS to assess urologic pathologies, addressing a significant barrier in providing quality treatment in this resource-limited setting.

## Ethics statement

The study was approved by the Institutional Review Board at University of California, San Francisco (UCSF) (IRB # 21-33758) and the Guyana Ministry of Health Institutional Review Board. Informational handouts were given to participants and verbal consent was given prior to participation.

## Study design

This was an observational cohort study of physicians-in-training (medical students and residents) at GPHC analyzing participant competency from a virtual POCUS education program. Reporting is based on the STROBE (Strengthening of Reporting of Observation Studies in Epidemiology) guidelines.

## Setting

This study was conducted in-person at GPHC and with virtual instruction from the UCSF from June to December 2021. This encompassed recruitment (June), the POCUS training phase (July–August), and the mentored implementation phase (September–December). During the training phase, students participated in a 1 hour combined Zoom lecture from UCSF researchers and in-person training at GPHC with their practicing urologists. Following, students were allotted 2 weeks to practice independently. In the mentored implementation phase, individual students were scheduled to perform guided urologic ultrasounds on patients under the guidance of a GPHC urologist.

## Participants

Eligibility criteria included physicians-in-training rotating through GPHC. The selection was on a volunteer basis, with 20 volunteers initially and 14 completing the entire program.

## Variables

Variables included participant POCUS knowledge, clinical skills, and satisfaction as assessed through written exams, an objective structured clinical exam (OSCE), a satisfaction survey, and the review of clinical scans.

## Data sources/measurement

After participants reviewed provided educational material [5,6], clinical knowledge was assessed through a written exam (Supplement 1) adapted from standardized questions developed by Wong et al [7]. Their clinical skills were tested in an OSCE administered by a GPHC urologist (B.R.) (Supplement 2). The OSCE was adapted from the Emergency Ultrasound Level 1 Triggered Assessment used for accreditation by the College of Emergency Medicine [7].

During the mentored implementation phase, students performed supervised POCUS scans as part of their diagnostic evaluation of patients at GPHC. They uploaded their images and interpretations without patient identifiers onto a secure online platform for document exchange. Their images were reviewed by a UCSF urologist (D.B.) for image quality and interpretation accuracy. A repeat written exam, reworded from the first but testing the same key points, was administered to observe clinical knowledge changes. Both written exams were graded by the same member of the team using the same criteria (O.N.).

All participants completed an anonymous satisfaction survey. Students were asked to rank the teaching course on a Likert scale (1=not satisfied at all, 5=very satisfied) and provide written feedback.

## Bias

Students self-selected to be a part of this education program and were not assigned or chosen. Grading and review were completed by the researchers. Bias in reviewing the written exam and images was mitigated by blinding reviewers. For the in-person OSCE, strict criteria were outlined on the grading form.

## Study size

A study size was not determined prior to the study and was determined by the number of available volunteers among rotating physicians-in-training at GPHC.

## Statistical methods

We performed descriptive statistics calculations for our data analysis.

## Key results

Our virtual POCUS education program aimed to introduce efficient diagnostic imaging in GPHC urology clinics by enhancing clinical knowledge and skills among physicians-in-training. Participants retained knowledge as indicated by overall improved averages on written exams, and they were able to perform scans to previously established standards, as evidenced by OSCE results. They were able to image urologic pathologies including obstructive uropathy and renal cysts.

## Main results

A total of 20 physicians-in-training volunteered to be part of this course, and data were analyzed for the 14 who completed the course. In the training phase, the trainees scored an average of 3.36/5 on the written exam. They scored highest on knowing which probe to use for transabdominal scanning (100%) and comparing tissue densities (100%), and lowest on differentiating urine from peritoneal fluid (50%), knowing the criteria for hydronephrosis (50%), and deciding when to use catheterization over bladder POCUS volume measurement (35.7%). All students earned 100% on the OSCE.

During the mentored implementation phase, students practiced scanning on clinic patients and repeated the written exam to reassess their knowledge. The average score was 3.57/5. They scored highest on knowing which probe to use for transabdominal scanning (100%) and the criteria for hydronephrosis (92.9%), and lowest on comparing tissue densities (57.1%), differentiating urine from peritoneal fluid (50%), and when to use catheterization over bladder POCUS volume measurement (57.1%).

**Fig. 1.**
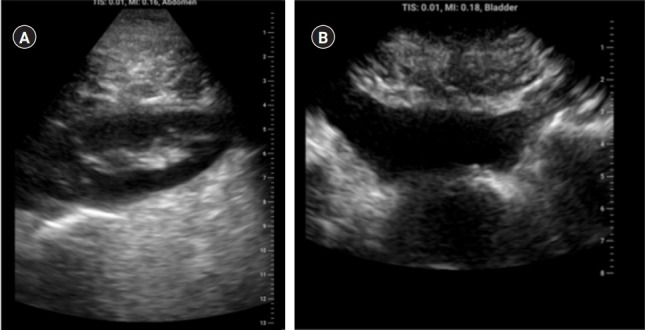
Mentored implementation phase student sample scans. (A) Normal kidney with no stones and no hydronephrosis. (B) Elevated residual urine in the setting of enlarged prostate.

All 14 students collected at least one image of the kidney and bladder, ranging from 2 to 4 studies per student, totaling 37 urologic studies. Students were able to image normal bladders and kidneys as well as pathologies such as enlarged prostates, hydronephrosis, and renal cysts (Fig. 1). In terms of satisfaction, all participants—including those who did not complete the study—provided anonymous feedback, and rankings averaged 3.9 on a Likert scale, indicating satisfaction. The most common feedback included (1) desire for increased emphasis on identifying pathologic conditions using POCUS and (2) time for hands-on practice. Raw data from the written exams, OSCE, satisfaction survey, and collected urologic studies are in Datasets 1–5.

## Interpretation

This pilot virtual POCUS education program introduced new imaging technology to participants while cultivating, maintaining, and applying their newfound knowledge in urology clinics. Looking at their clinical knowledge, compared to the first written exam, they improved in knowing the criteria for hydronephrosis and when to use catheterization over bladder POCUS measurement. They were weaker in identifying tissue appearance, and they performed similarly on knowing which probe to use for transabdominal scanning and differentiating bladder from peritoneal fluid. Given the slight improvement in average exam scores, students were able to maintain their knowledge through the course. In terms of patient scans, students were generally able to collect images of normal anatomy and some pathology, including enlarged prostates and bladder distention. From participant feedback, the program could improve from better focus on diagnostics and opportunities for training.

## Comparison with previous studies

This is the first known study to establish a POCUS education program in a LMIC that is specific to urology. Ultrasound teaching programs in LMICs have generally been in cardiology and gastroenterology and have shifted towards educating non-physicians including midwives and community health workers [8]. Similarly, we focused our study on physicians-in-training to integrate POCUS knowledge early in training. Our study also demonstrated POCUS knowledge and clinical competency through our program. This aligns with previous studies in LMICs that involved specialty-tailored POCUS teaching that contributed to provider skill sets and diagnostic ability [2,3]. Recent literature has also shown the feasibility of using telecommunications to provide POCUS training across geographic divides. While our study integrated virtual lectures and exams, others have experimented with tele-ultrasonography, messaging systems, and teleconsultation [9,10]. In our increasingly transcontinental landscape, virtual and collaborative approaches to imaging diagnostics show potential in providing improved health education in low-resource settings.

## Limitations/generalizability

In-person training and practice sessions were limited and sometimes delayed given restrictions and changes associated with the coronavirus disease 2019 pandemic. For the evaluative components, reviewers were aware of the objectives of the study, although strict criteria alleviated bias. Our cohort was small and composed of self-motivated volunteers exclusively from GPHC. The findings in this group may not be generalizable to other LMIC settings. Moreover, this was a single site study, and a similar program at another institution may have varying results depending on resources, institutional support, and educational opportunities.

## Suggestions

Future studies may look at quality control measures in ultrasound assessment and training, methods for long-term retention, and impacts on urologic care, including use of open procedures.

## Conclusion

This preliminary results suggest that our virtual program is satisfactory to participants and provides a feasible method to teach clinical skills through remote learning across international borders. Since we did not take a baseline knowledge and skills assessment before starting the education program, it is impossible to confirm an improvement in competency from this program. This pilot study better represents the feasibility of initiating a training program in a low-resource setting. It demonstrates the potential of global collaborations using virtual didactics for POCUS education.

## Figures and Tables

**Figure f2-jeehp-20-11:**
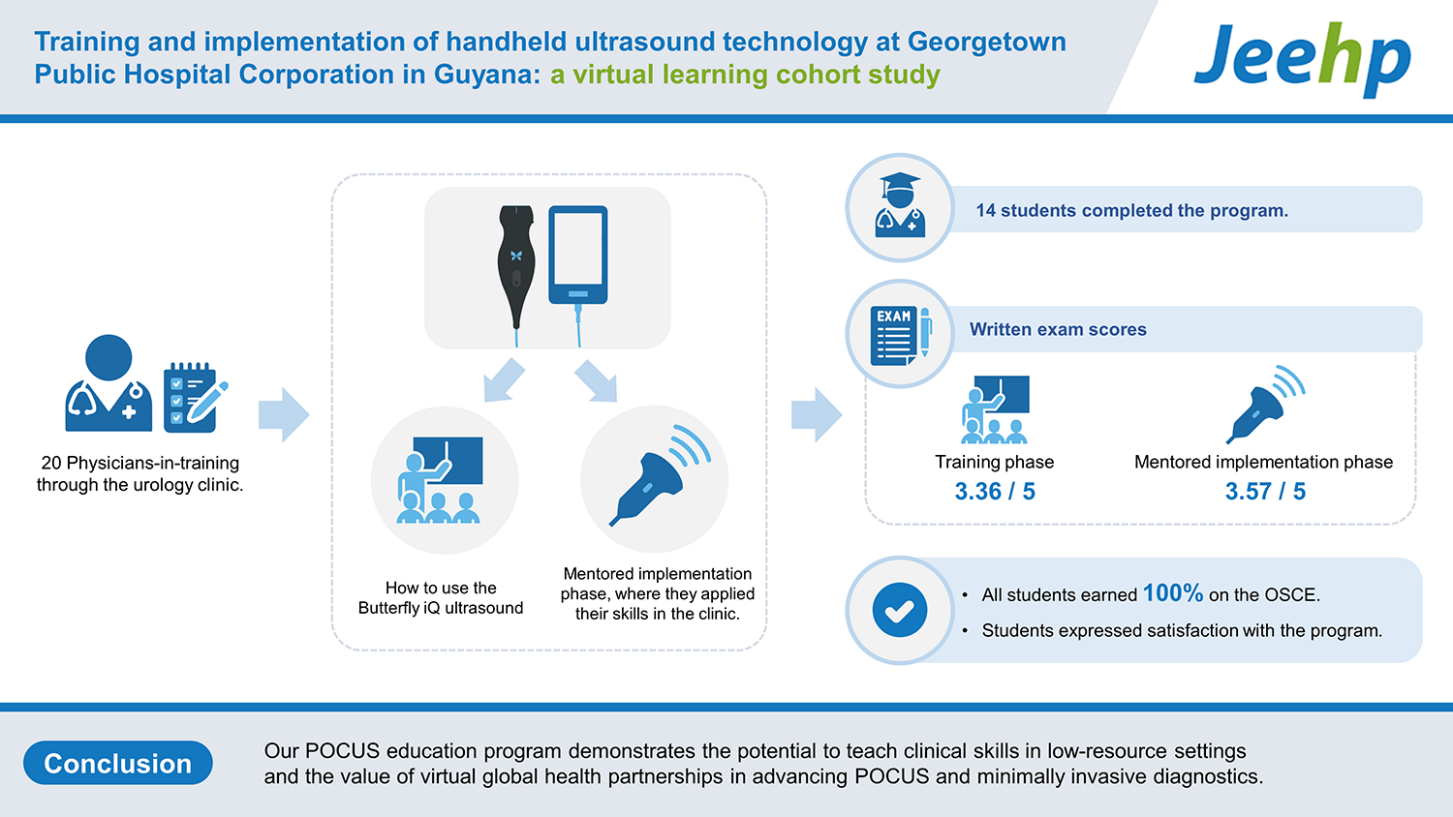


## Footnotes

**Data availability**
Data files are available from Harvard Dataverse: https://doi.org/10.7910/DVN/3CDPME Dataset 1. The data file contains the written exam results from the training phase.

Dataset 2. The data file contains the written exam results from the mentored implementation phase.

Dataset 3. The data file contains the objective structured clinical exam results from the end of the training phase.

Dataset 4. The data file contains the satisfaction survey results.

Dataset 5. The data file contains the student urologic imaging studies and interpretations from the mentored implementation phase.

## Supplementary materials

Supplementary files are available from Harvard Dataverse: https://doi.org/10.7910/DVN/3CDPME

Supplement 1. Written exam.

Supplement 2. Objective structured clinical exam.

Supplement 3. Audio recording of the abstract.

## References

[1] Mollura DJ, Soroosh G, Culp MP, RAD-AID Conference Writing Group (2017). 2016 RAD-AID Conference on International Radiology for Developing Countries: Gaps, Growth, and United Nations Sustainable Development Goals. J Am Coll Radiol.

[2] Henwood PC, Mackenzie DC, Rempell JS, Douglass E, Dukundane D, Liteplo AS, Leo MM, Murray AF, Vaillancourt S, Dean AJ, Lewiss RE, Rulisa S, Krebs E, Raja Rao AK, Rudakemwa E, Rusanganwa V, Kyanmanywa P, Noble VE (2016). Intensive point-of-care ultrasound training with long-term follow-up in a cohort of Rwandan physicians. Trop Med Int Health.

[3] Tafoya CA, Tafoya MJ, Osei-Ampofo M, Oteng RA, Becker TK (2017). Sustainable resuscitation ultrasound education in a low-resource environment: the Kumasi experience. J Emerg Med.

[4] Metzler I, Bayne D, Chang H, Jalloh M, Sharlip I (2020). Challenges facing the urologist in low- and middle-income countries. World J Urol.

[5] Jensen JA (2007). Medical ultrasound imaging. Prog Biophys Mol Biol.

[6] Kim CL (2020). Ultrasound principles & instrumentation: orientation & imaging planes [Internet]. https://www.youtube.com/watch?v=j7X5-KMyEcA.

[7] Wong I, Jayatilleke T, Kendall R, Atkinson P (2011). Feasibility of a focused ultrasound training programme for medical undergraduate students. Clin Teach.

[8] Becker DM, Tafoya CA, Becker SL, Kruger GH, Tafoya MJ, Becker TK (2016). The use of portable ultrasound devices in low- and middle-income countries: a systematic review of the literature. Trop Med Int Health.

[9] Bansal M, Singh S, Maheshwari P, Adams D, McCulloch ML, Dada T, Sengupta SP, Kasliwal RR, Pellikka PA, Sengupta PP, VISION-in-Tele-Echo Study Investigators (2015). Value of interactive scanning for improving the outcome of new-learners in transcontinental tele-echocardiography (VISION-in-Tele-Echo) study. J Am Soc Echocardiogr.

[10] Chamadol N, Laopaiboon V, Srinakarin J, Loilome W, Yongvanit P, Thinkhamrop B, Khuntikeo N (2017). Teleconsultation ultrasonography: a new weapon to combat cholangiocarcinoma. ESMO Open.

