# Supplementary material for: Training and implementation of handheld ultrasound technology at Georgetown Public Hospital Corporation in Guyana: a virtual learning cohort study
Source: J Educ Eval Health Prof. 2023 Apr 4;20:11. doi: 10.3352/jeehp.2023.20.11 (PMC11009011; doi:10.3352/jeehp.2023.20.11)
Supplement: Supplementary file 5 [file jeehp-20-11-dataset5.docx]

Student Urologic Image Studies and Interpretations

Note: Some students included their own names on images. Their names have been blocked for privacy. Numbers indicate start of new image set from the same student. Students provided interpretations differently (on the image or separate file).


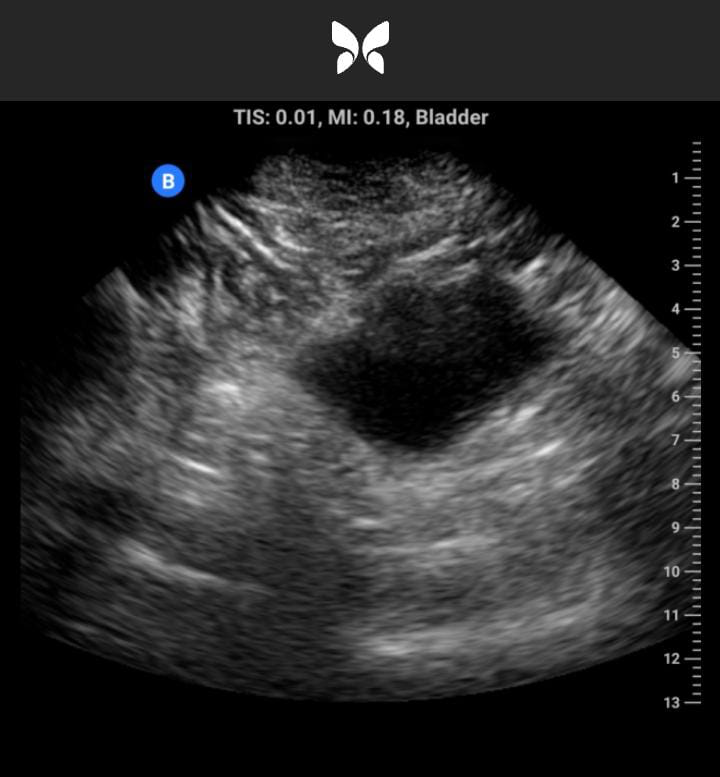


Transverse view of the bladder of a 59 y/o male pt. with PMHx of HTN and an MI with foley's catheter in situ. The full extent of the bladder could not be visualized since it was almost empty and actively being drained. The bladder was anechoic containing urine. No masses, stones, wall thickening, diverticula nor signs of urinary retention ie. overly distended bladder. The bladder appears normal. The prostate gland was not appreciated.


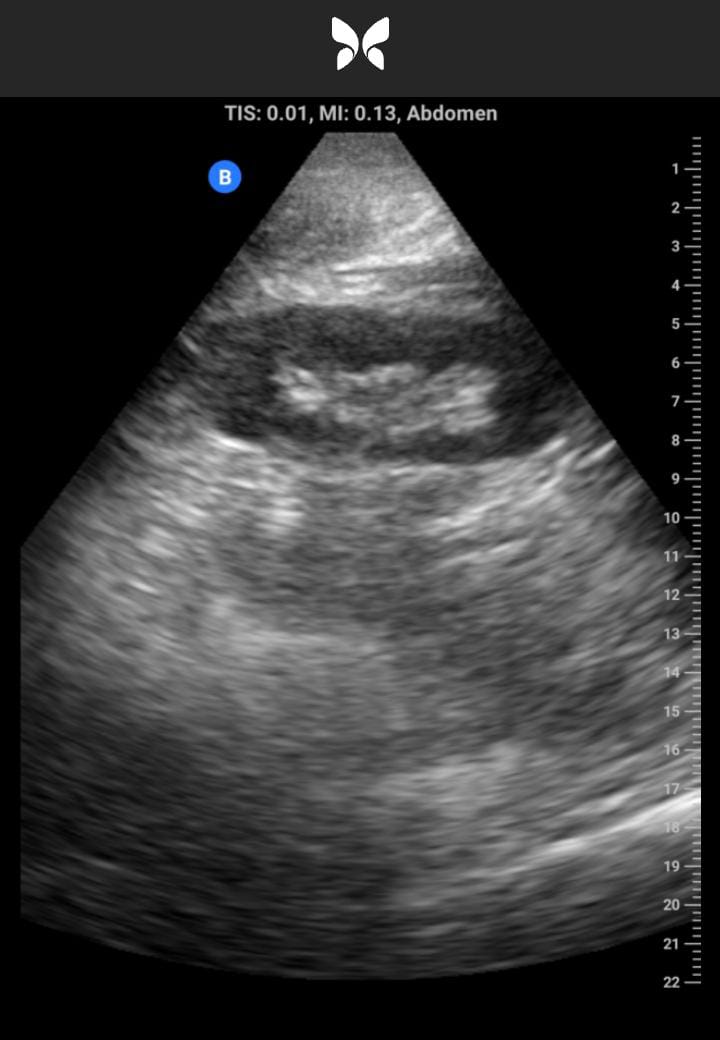


Longitudinal view of the right kidney of a 59 y/o with PMHx of HTN and an MI. The cortex and renal pelvis are well delineated. There was no obstruction from stones, no cysts, abscess nor tumour in the kidney. The borders appear regular with no signs of enlargement or hydronephrosis. The kidney appears normal. The liver can be visualized at the superior pole.


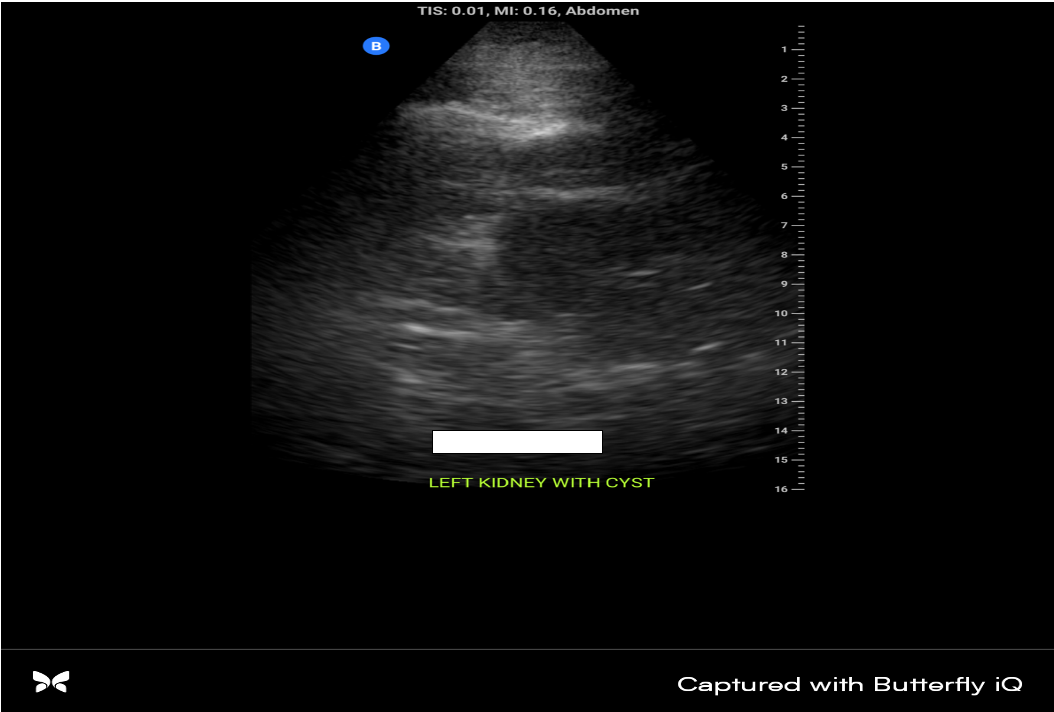


58 Y/o male patient with PMH of Renal Canaliculi presented with intermittent flank Pain. On Ultrasound of left kidney (Longitudinal view), the image shows an anechoic region to the Inferior pole of the kidney, which is suggestive of a cyst. There are no obvious canaliculi. Uncertain about any presence of hydronephrosis, renal pelvis or calyces dilatation.


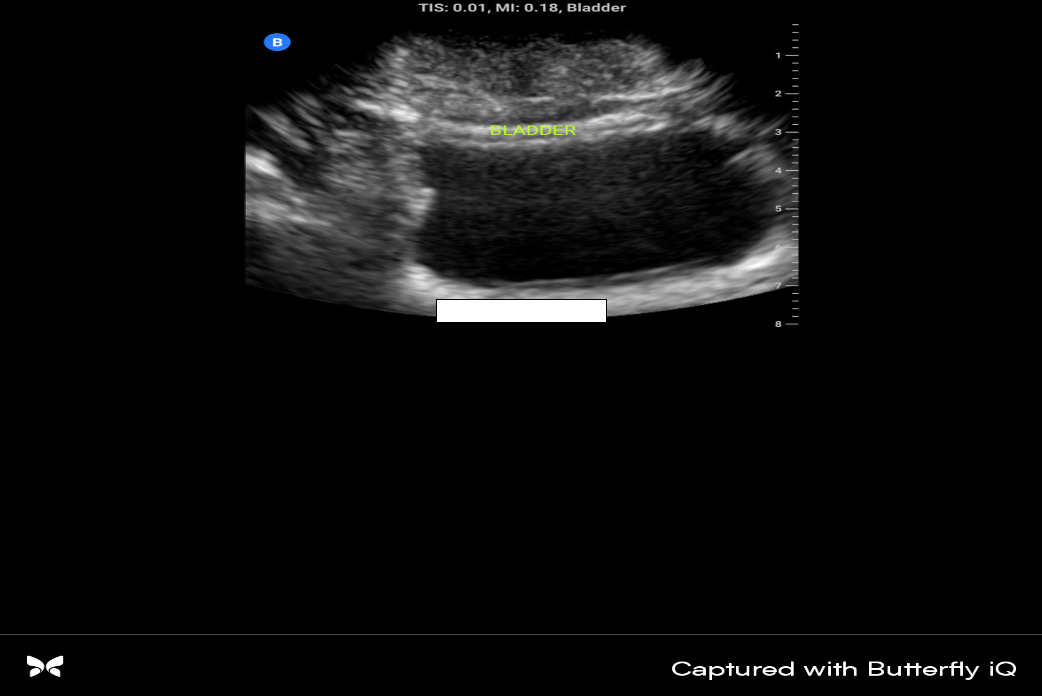


58 y/o male patient with PMH of Renal Canaliculi presented with intermittent flank pain. On Ultrasound of Bladder, There is approximately 210mls of urine in the bladder of which has a relatively uniform anechoic area. There is no obvious wall thickening or mass within the bladder. The findings are consistent with a normal bladder.


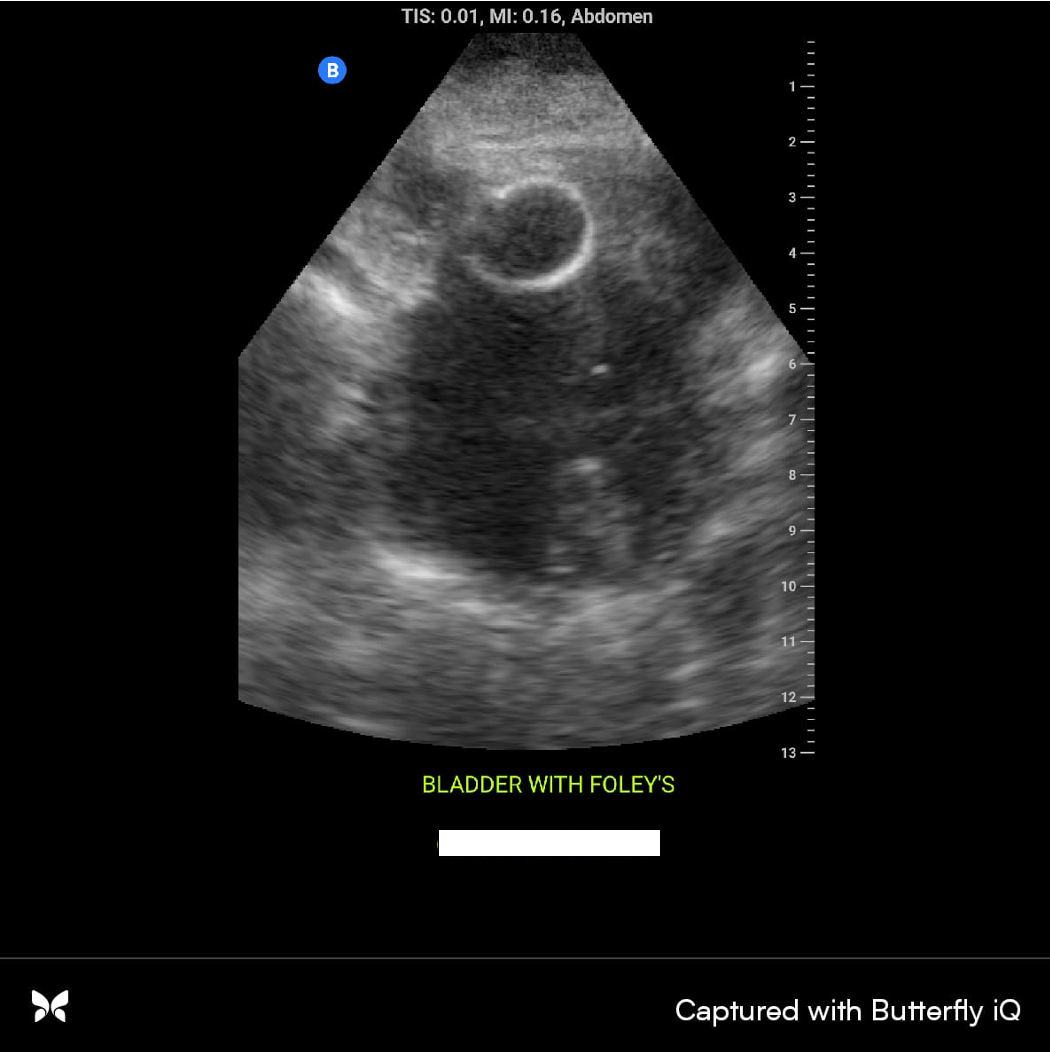


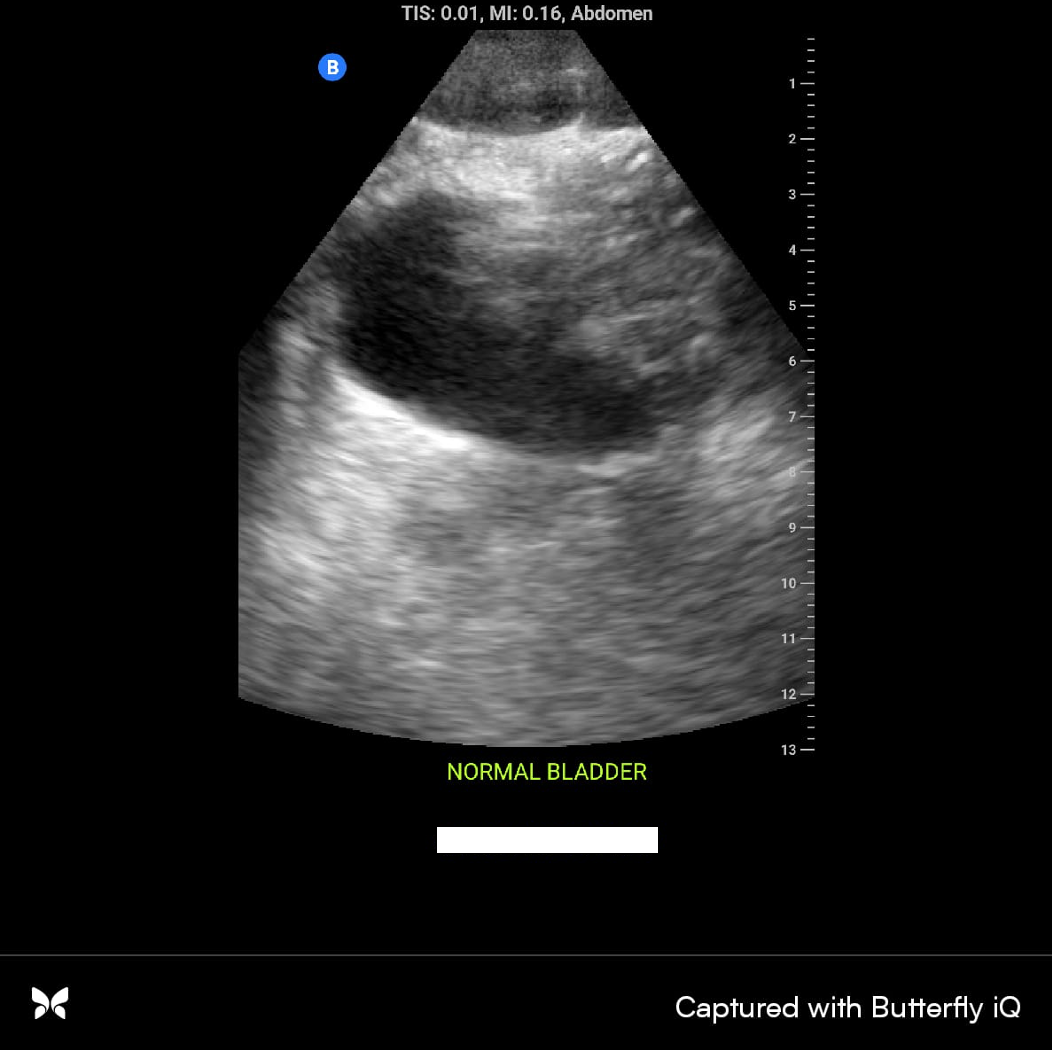


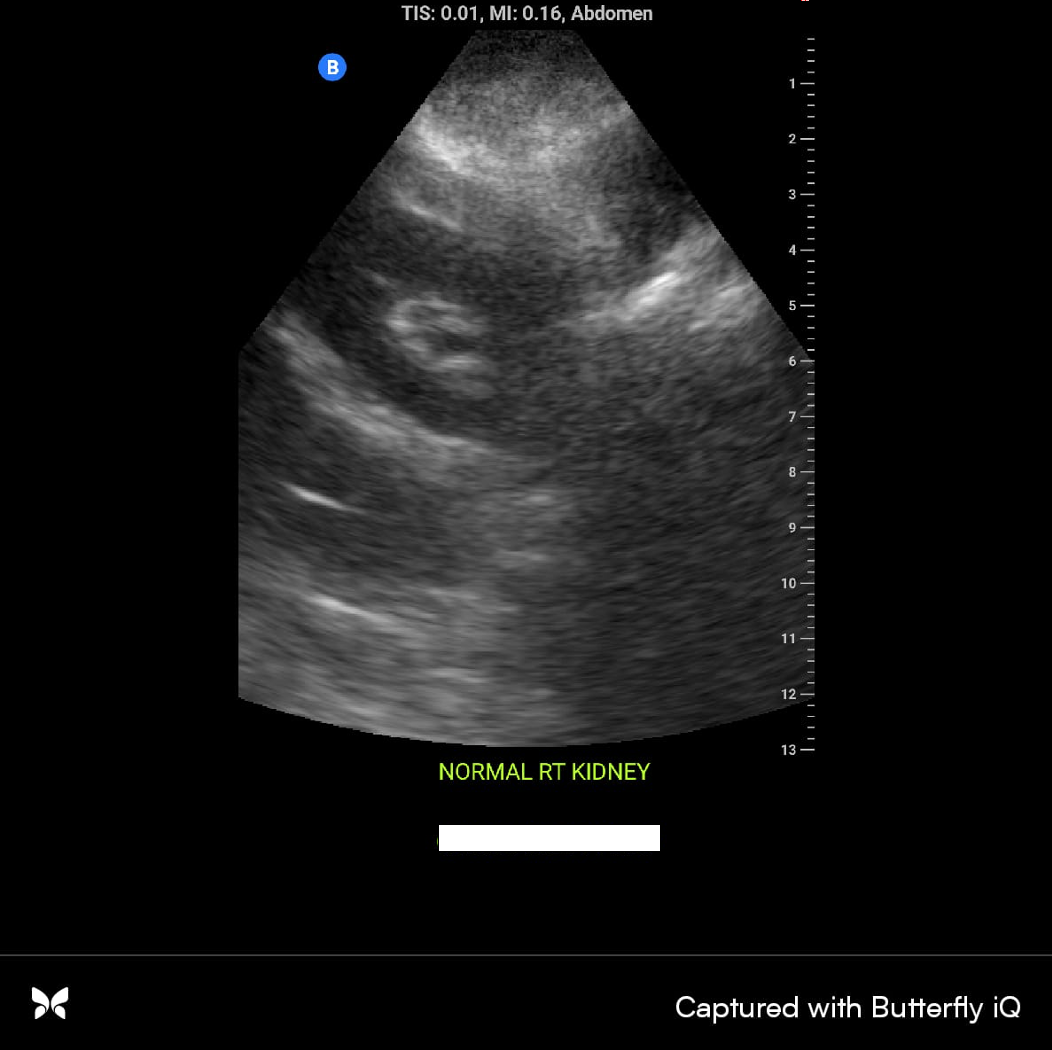


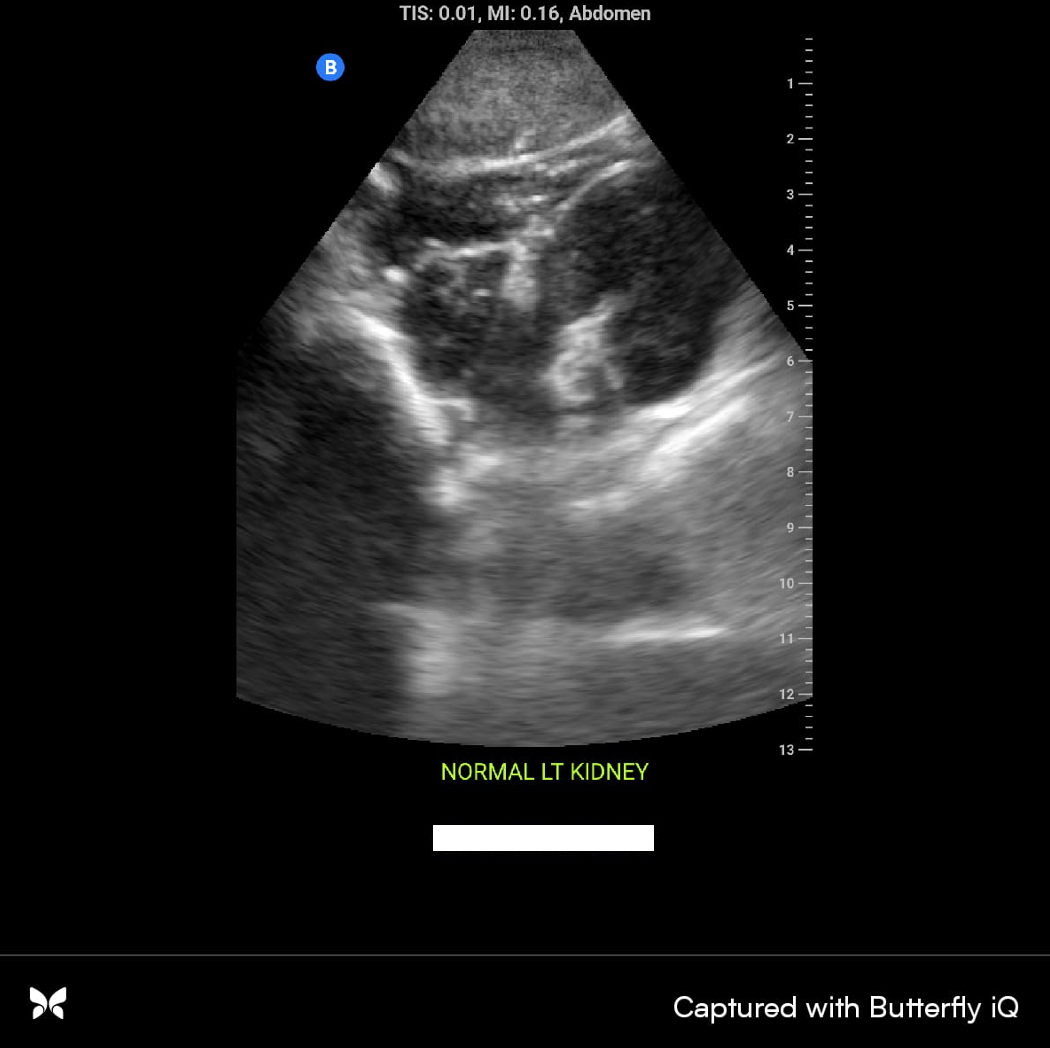


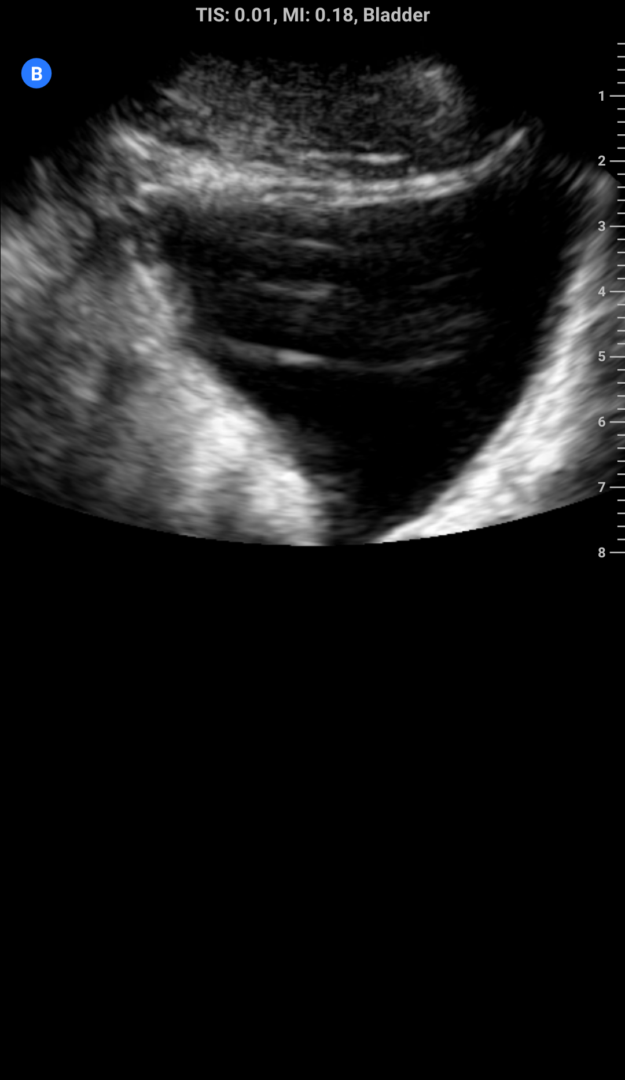


26-year-old male with no significant past medical history. Sagittal view on ultrasound shows anechoic bladder with urine and no obvious mass lesions on the bladder wall. Findings consistent with a normal bladder.


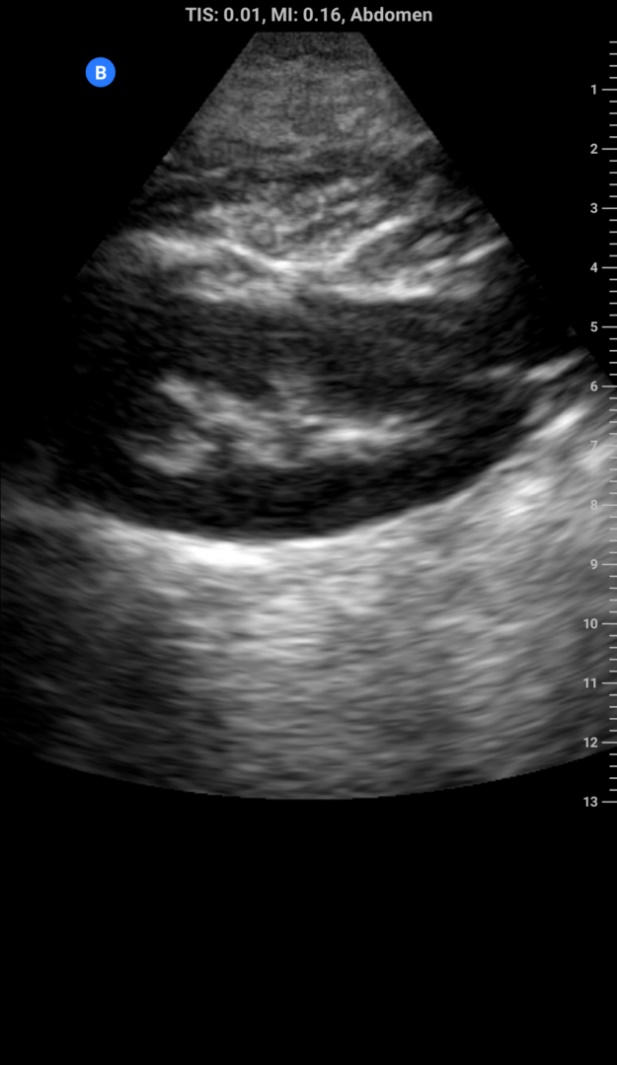


25-year-old male with no significant past medical history. Ultrasound of Kidney (longitudinal view) reveals no obvious dilatation of the renal pelvis and calyces. There is no appreciable calculi or perinephric fluid collections. Findings consistent with a normal kidney.


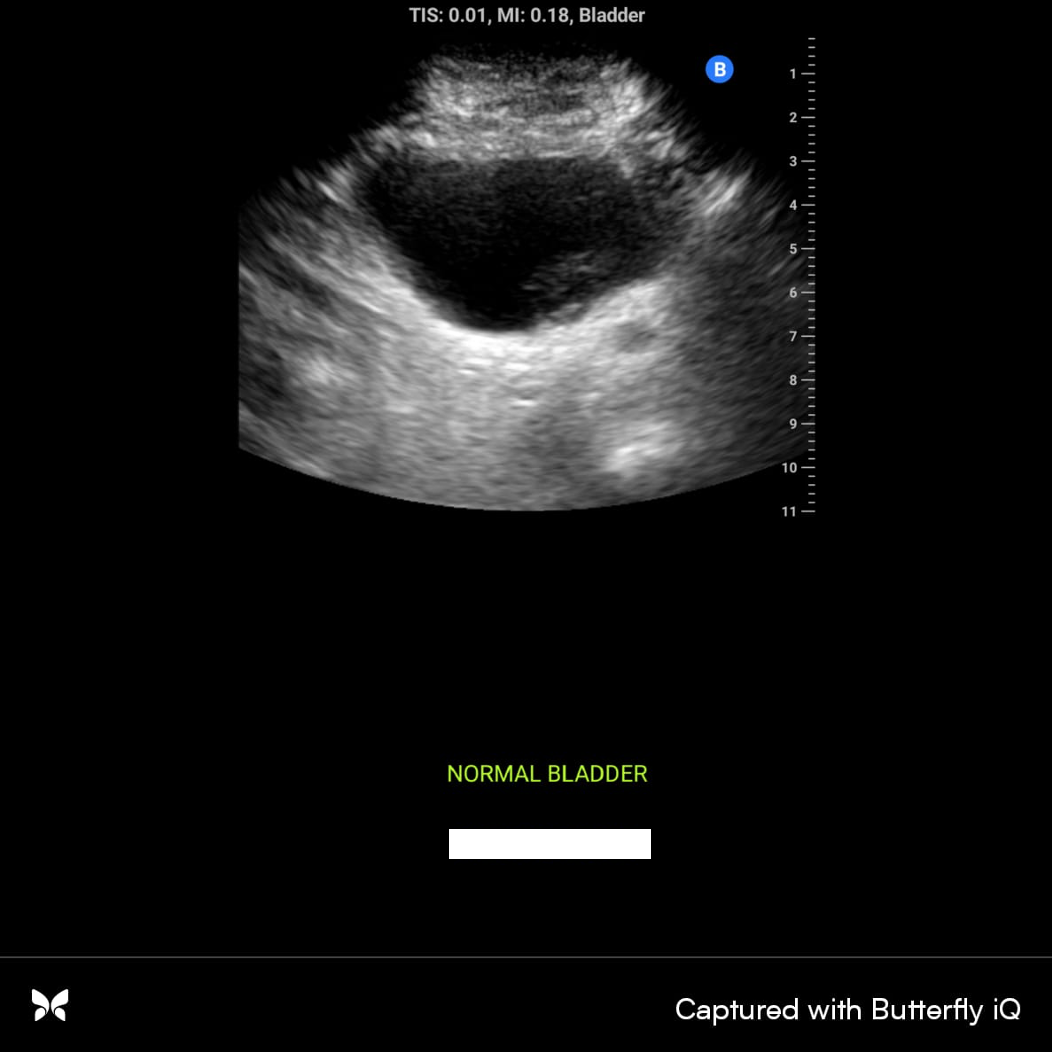


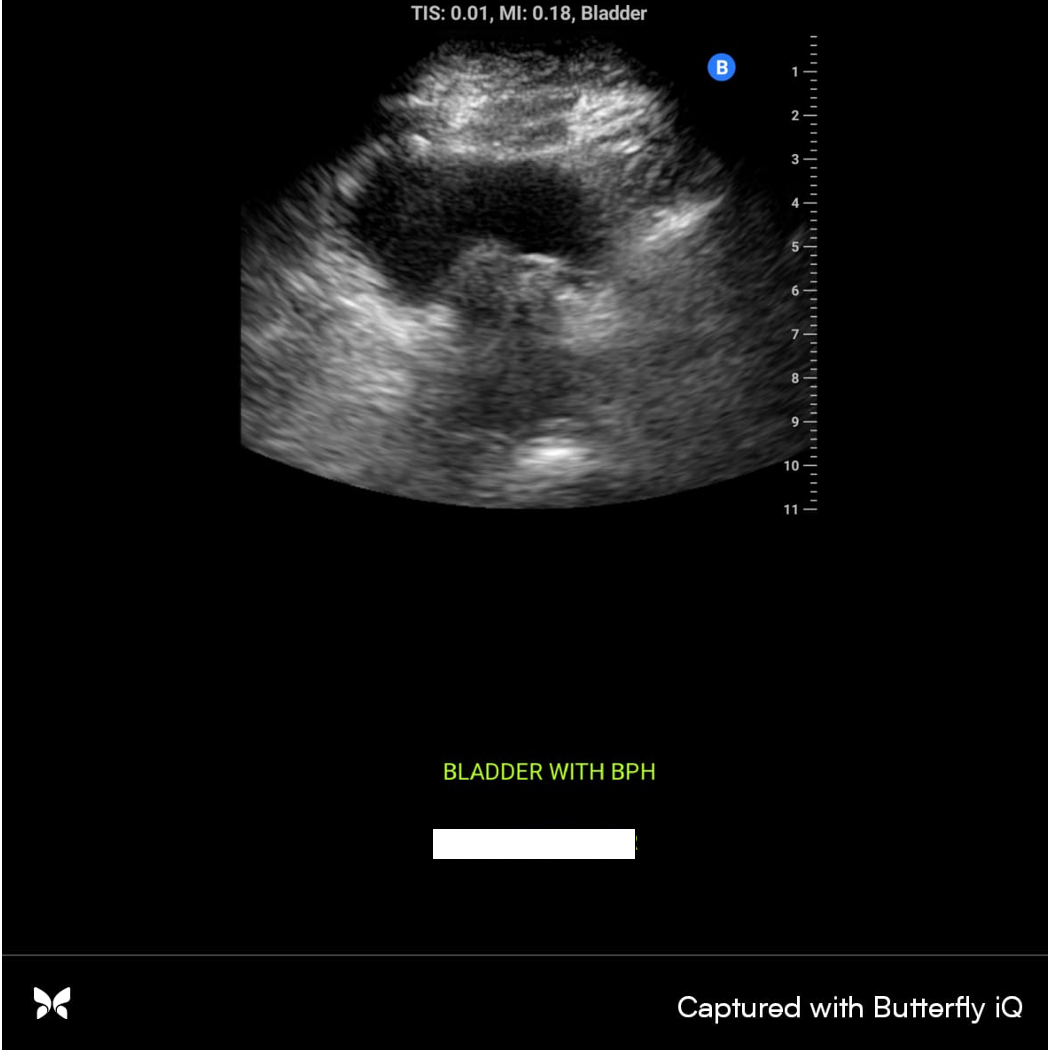


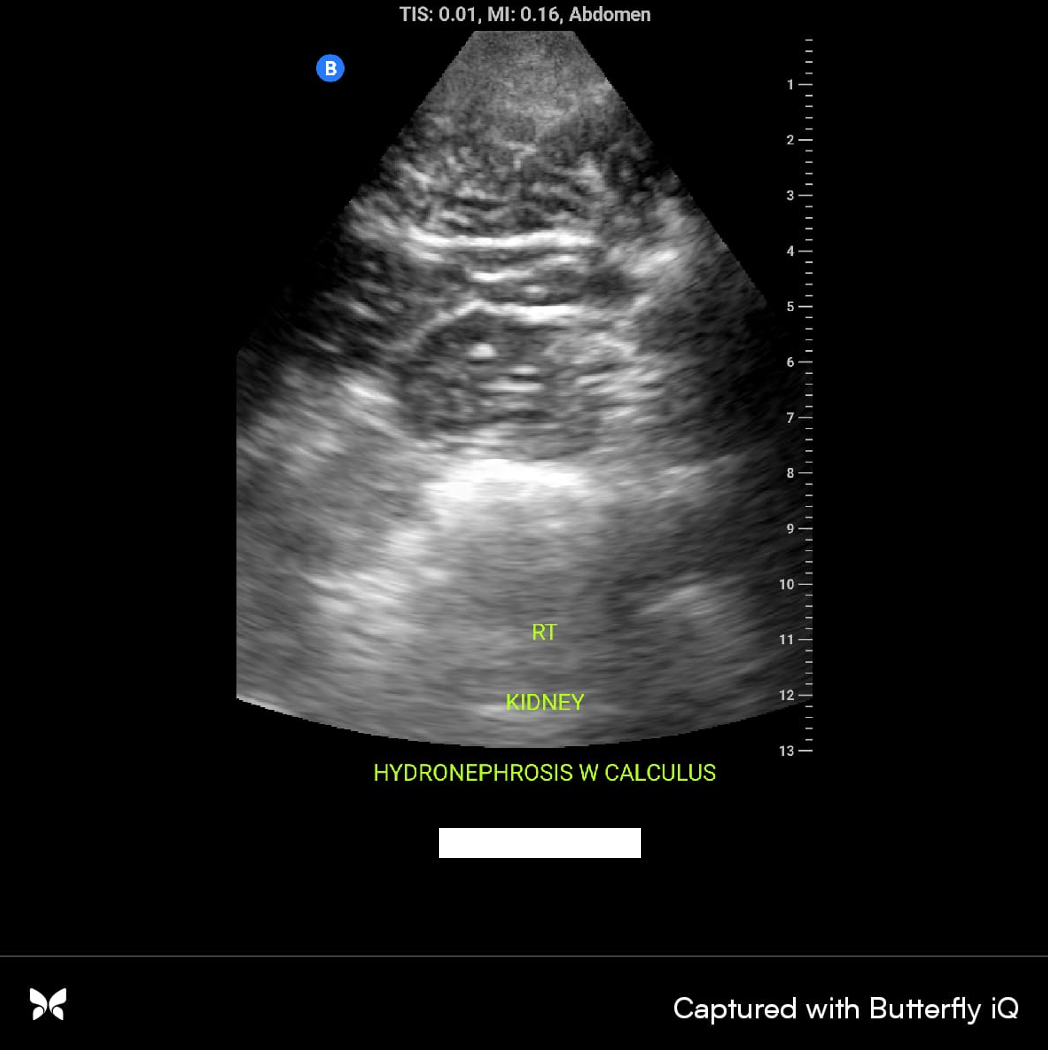


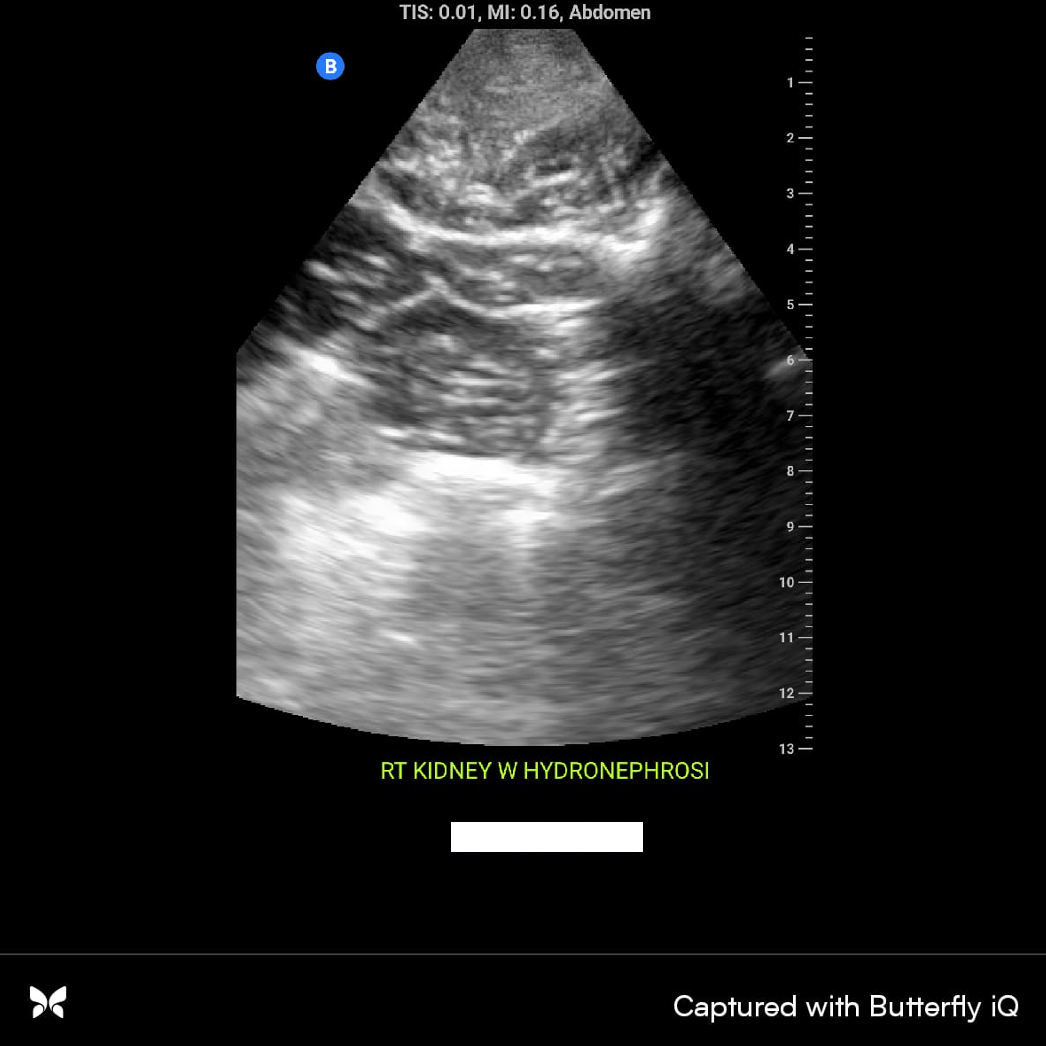


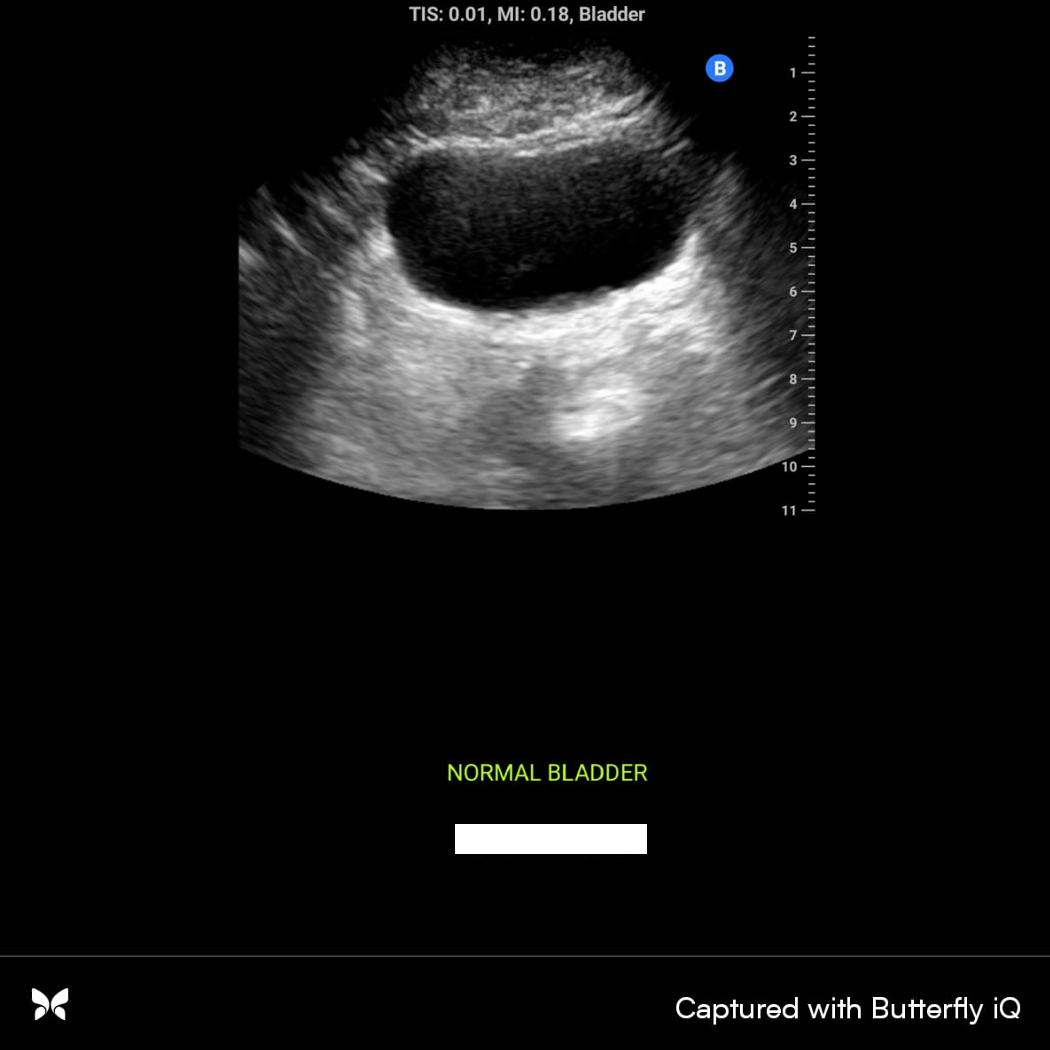


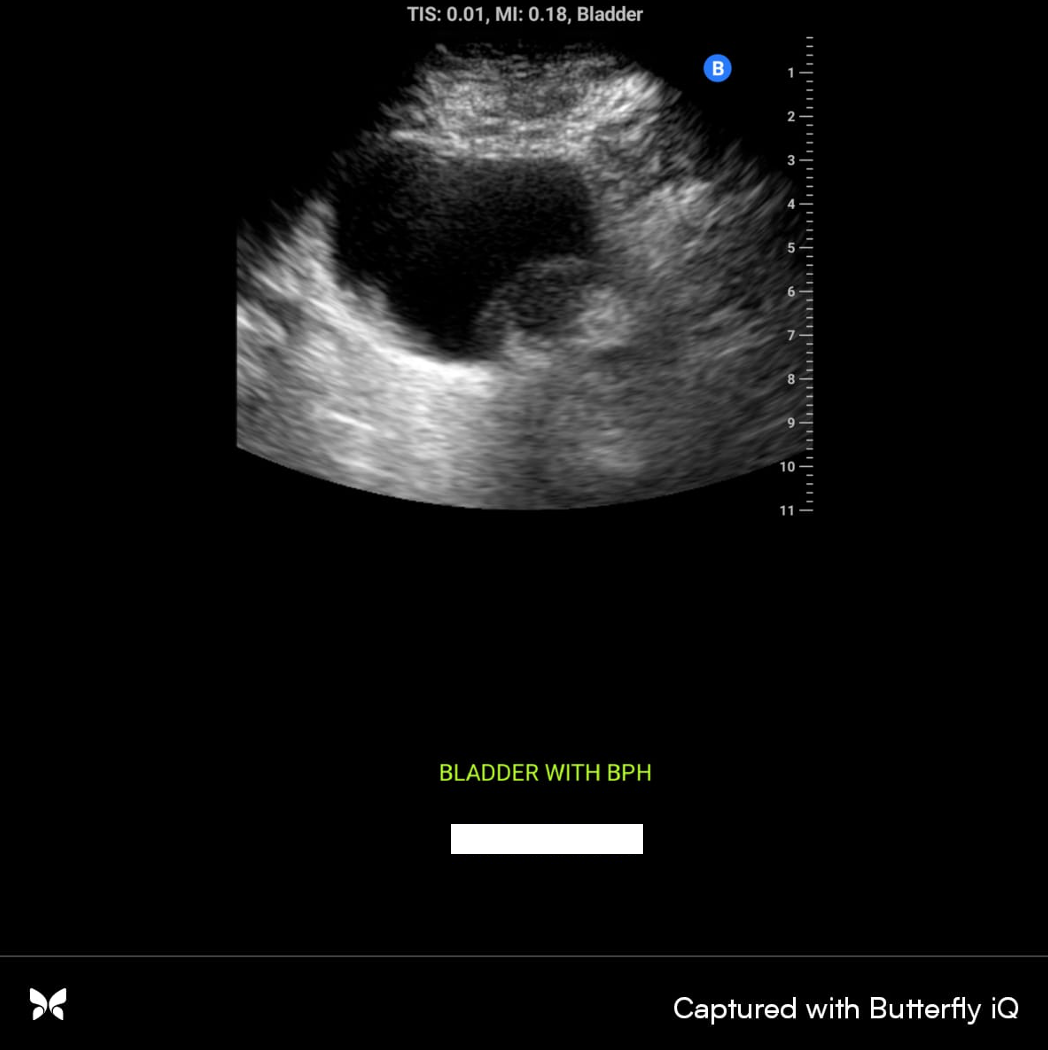


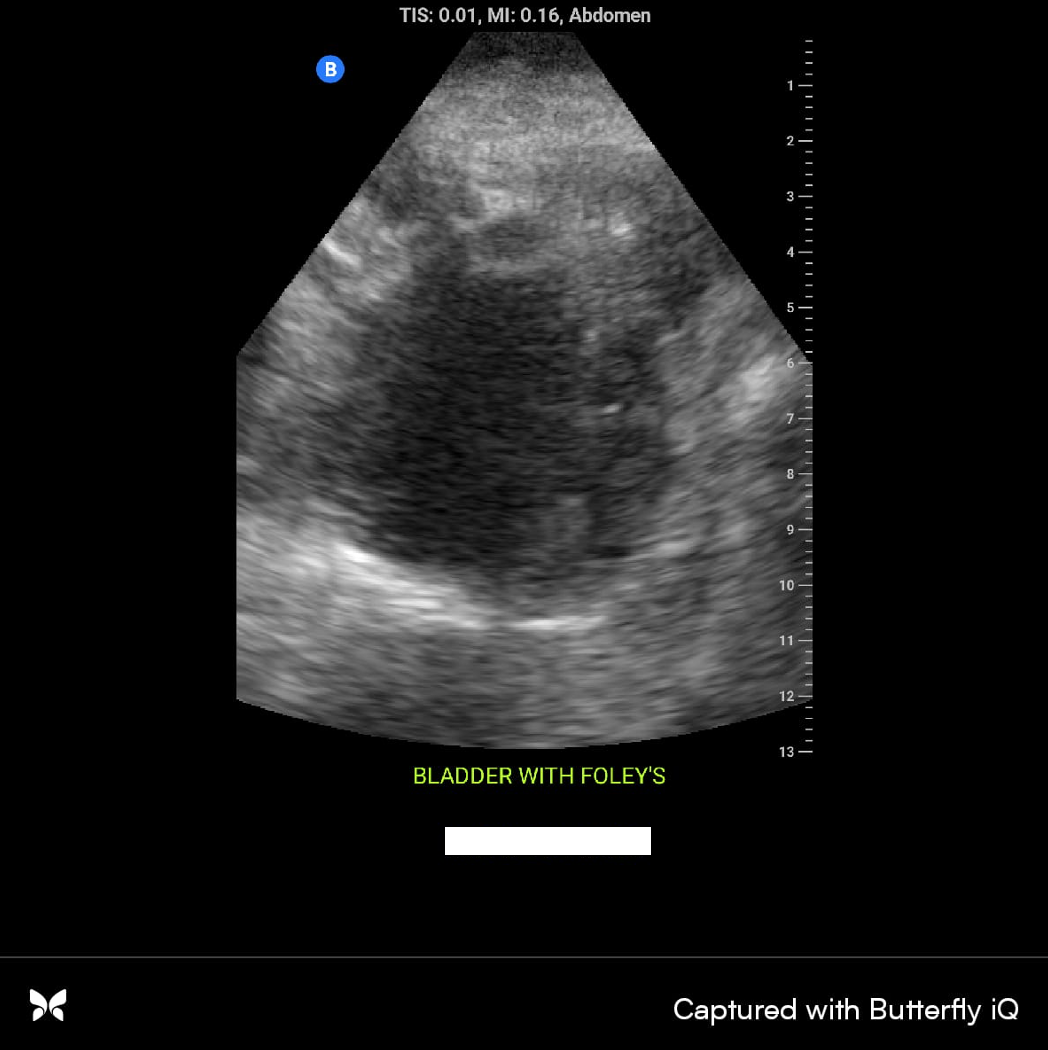


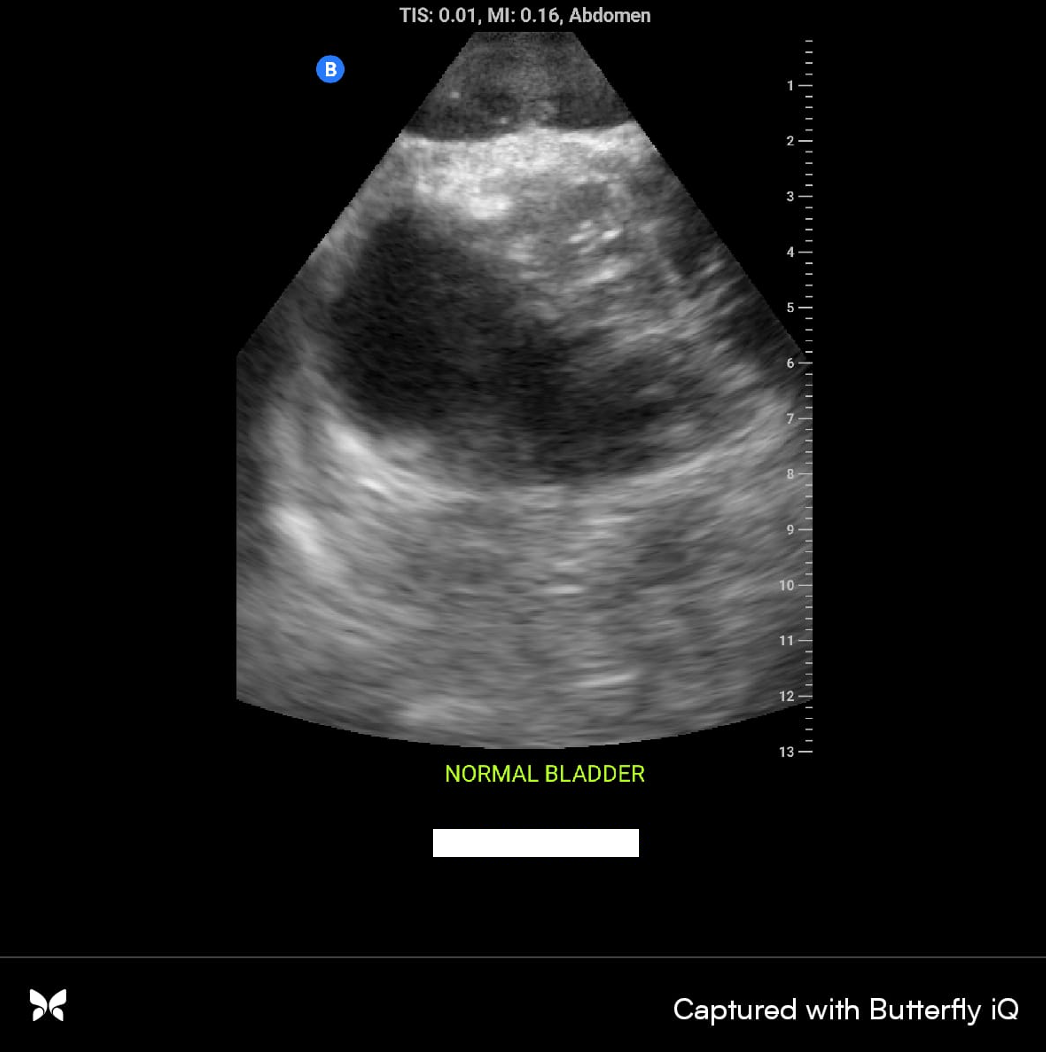


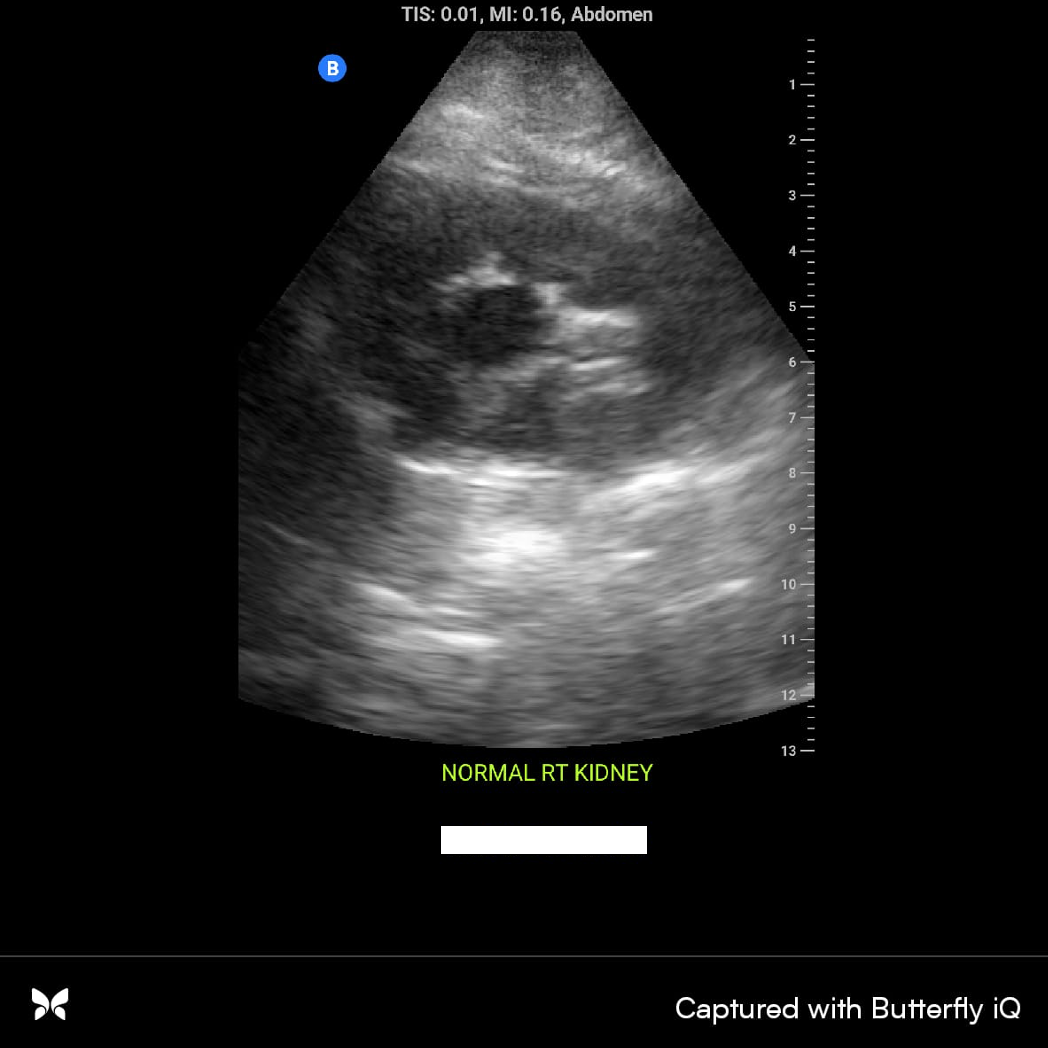


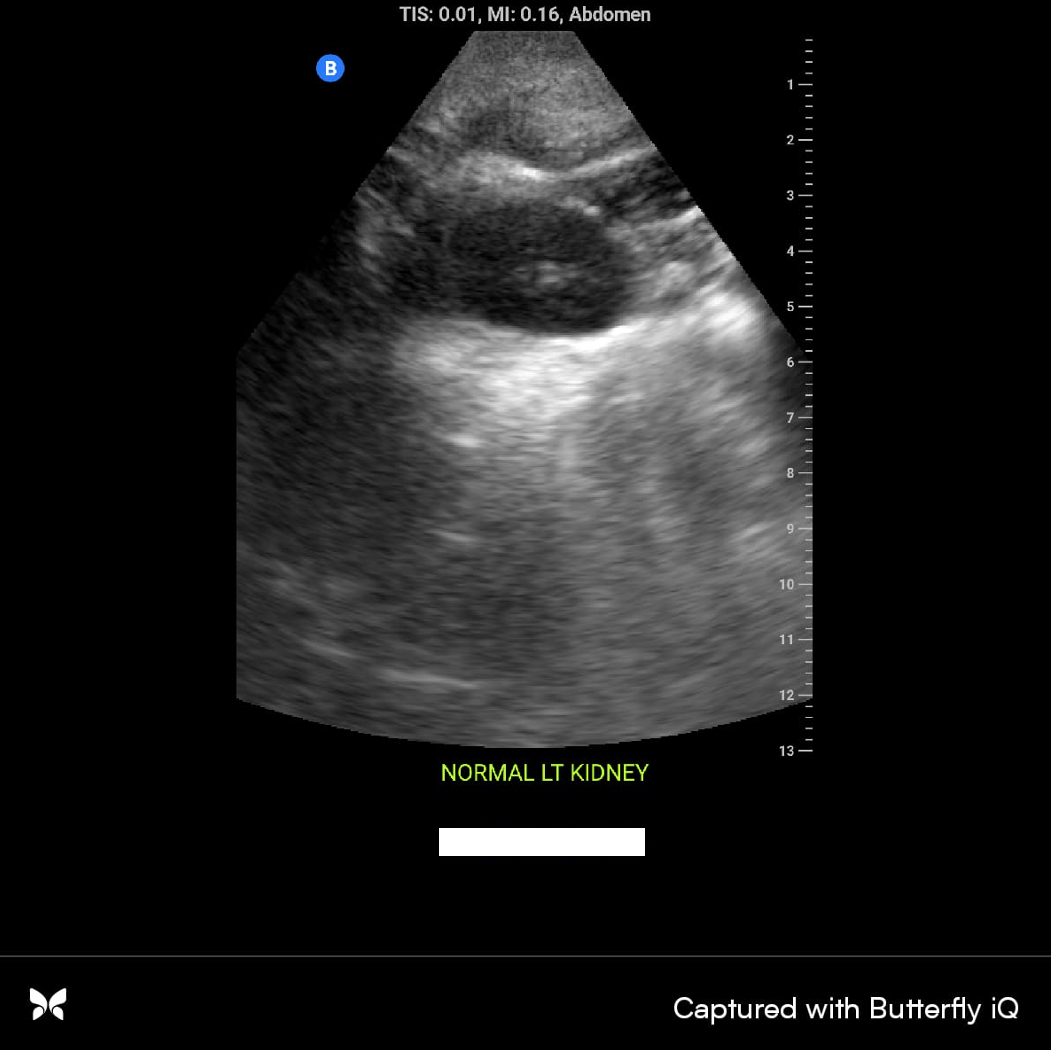


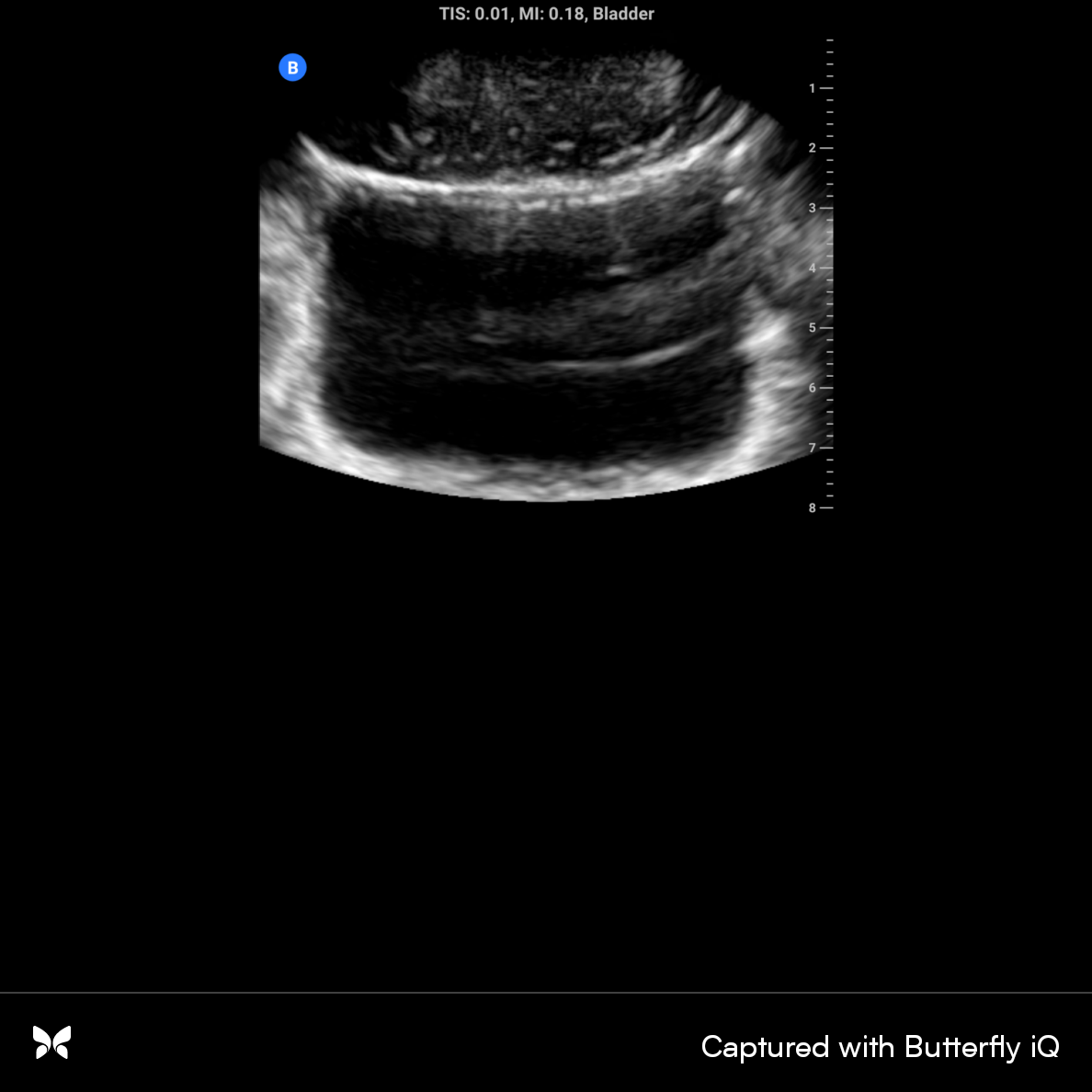


Transverse section of Bladder of 24 Year old male. Bladder is distended. No signs of wall thickening, hyperechoic bladder stones or echogenic masses visualised.


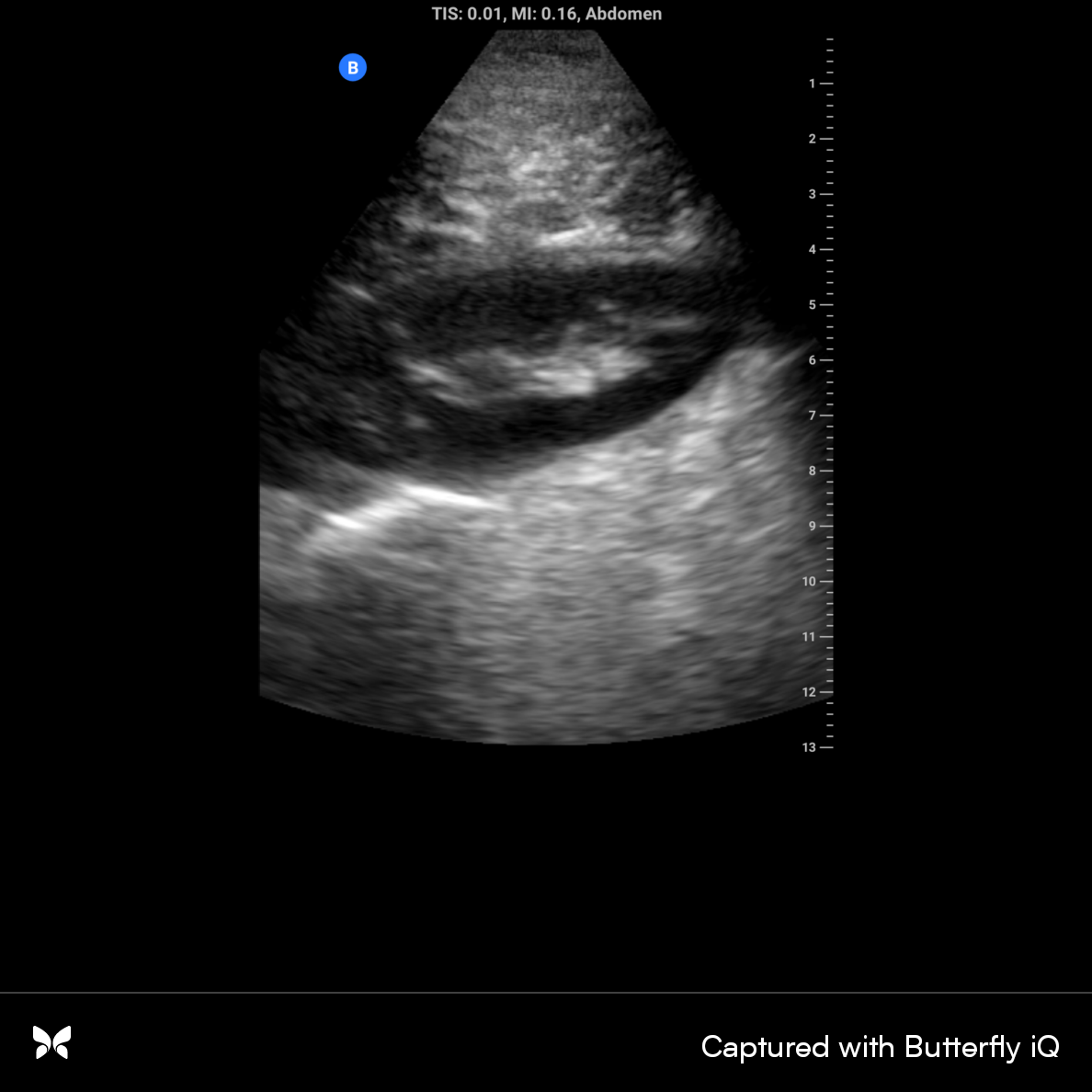


Longitudinal View of R kidney of 24-year-old male with history of renal calculi. Kidney anatomical contour and size appear to be normal, no calculi appreciated, hydronephrosis or peritoneal collections appreciated.


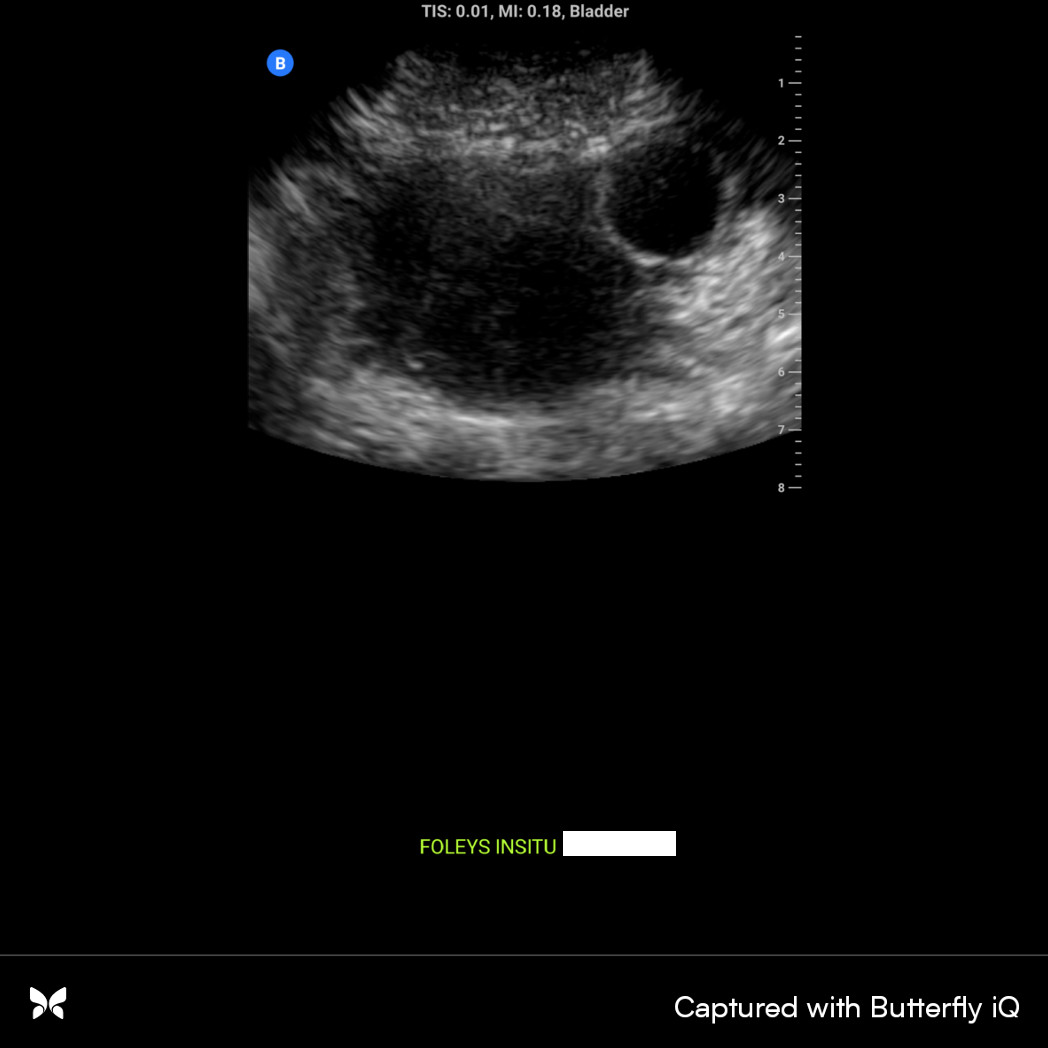


Bladder of a 73 year old male patient with a 6 year history of Benign Prostatic Hypertrophy.

Foley’s catheter can be appreciated as hyperechoic ring near the wall of the bladder.


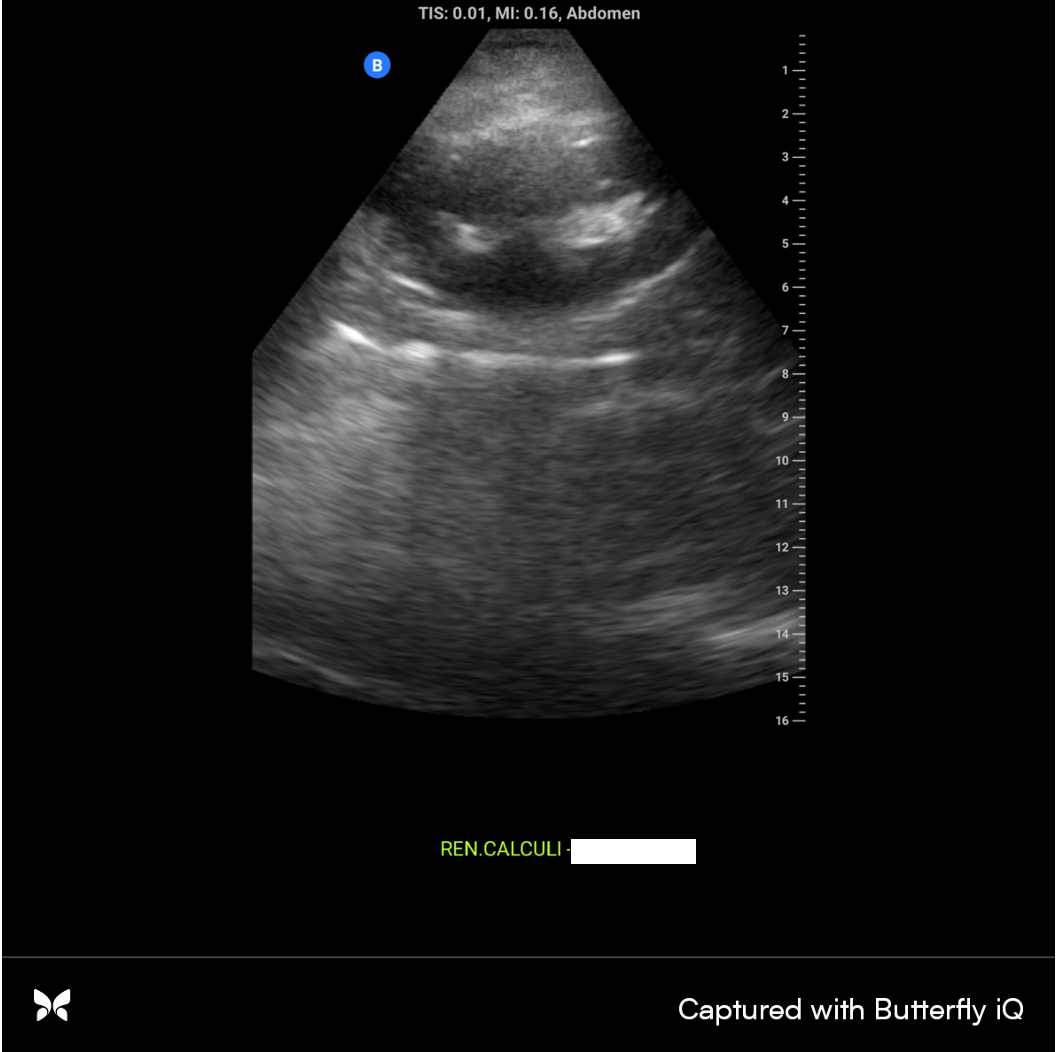


Right kidney of a 49 year old female patient with a 6 month history of ‘stabbing’ back pain

Renal calculi can be appreciated as echogenic area near the medulla. No hydronephrosis was detected.


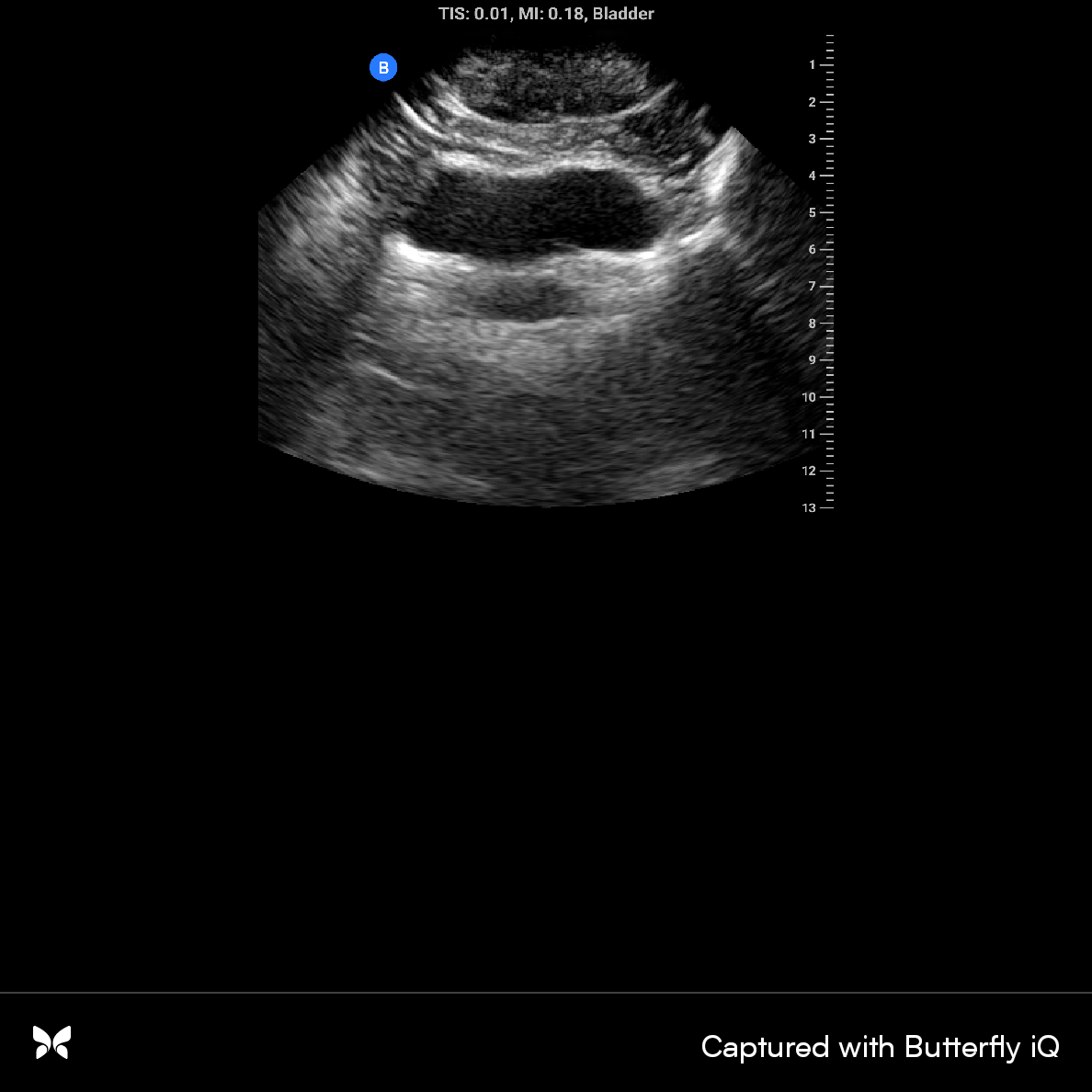


26-year-old male presented with right-sided flank pain. Ultrasound of bladder (axial view) reveals uniformly anechoic bladder with urine. No mass lesions appreciated within the bladder or in the wall. Prostate was appreciated inferior to the bladder. Findings consistent with a normal bladder.


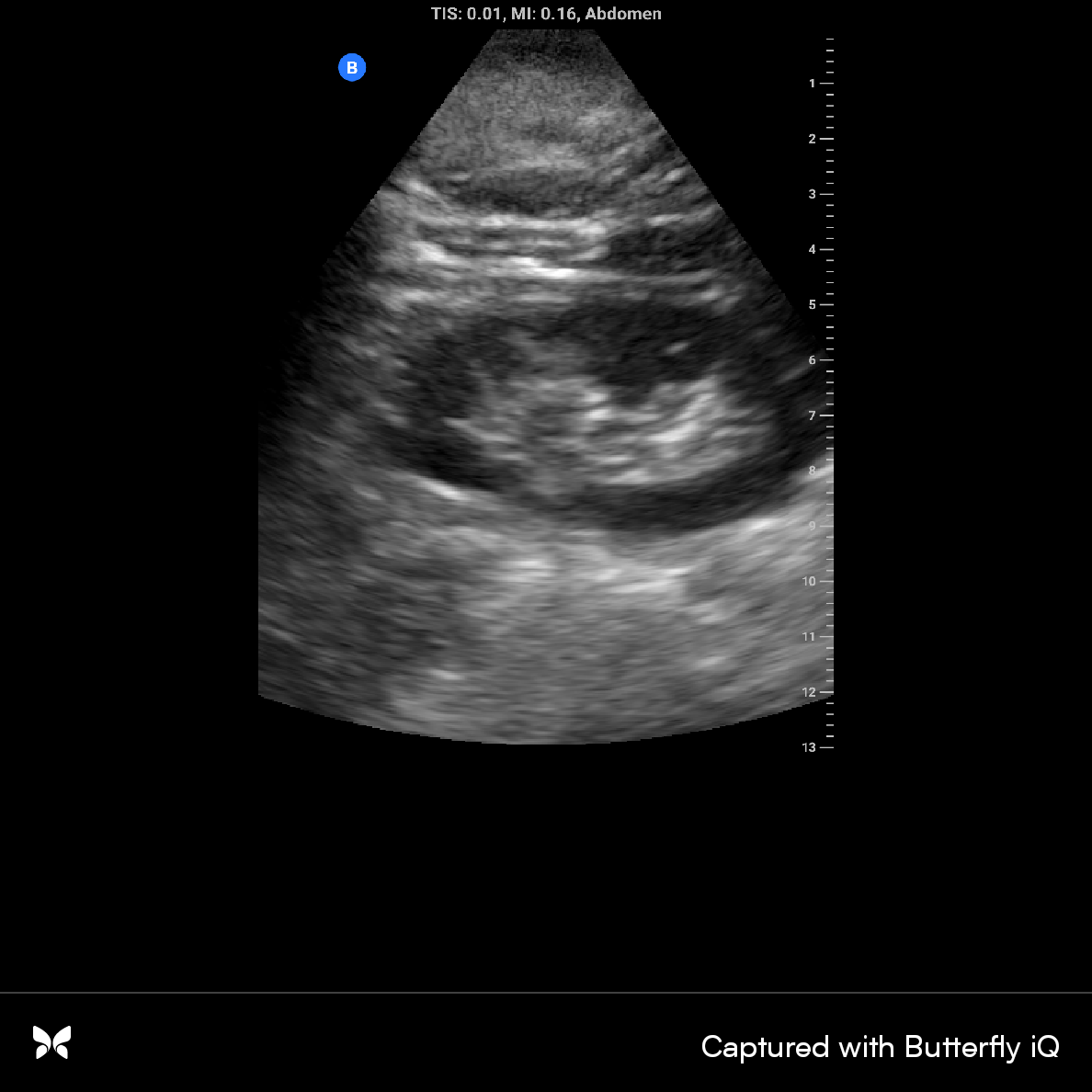


26-year-old male presented with right-sided flank pain. Ultrasound of kidney (longitudinal view) shows hypogenic cortex compared to liver parenchyma above. No dilatation of the renal pelvis or calyces were appreciated. No mass lesions including renal calculi were appreciated. Findings consistent with a normal right kidney.


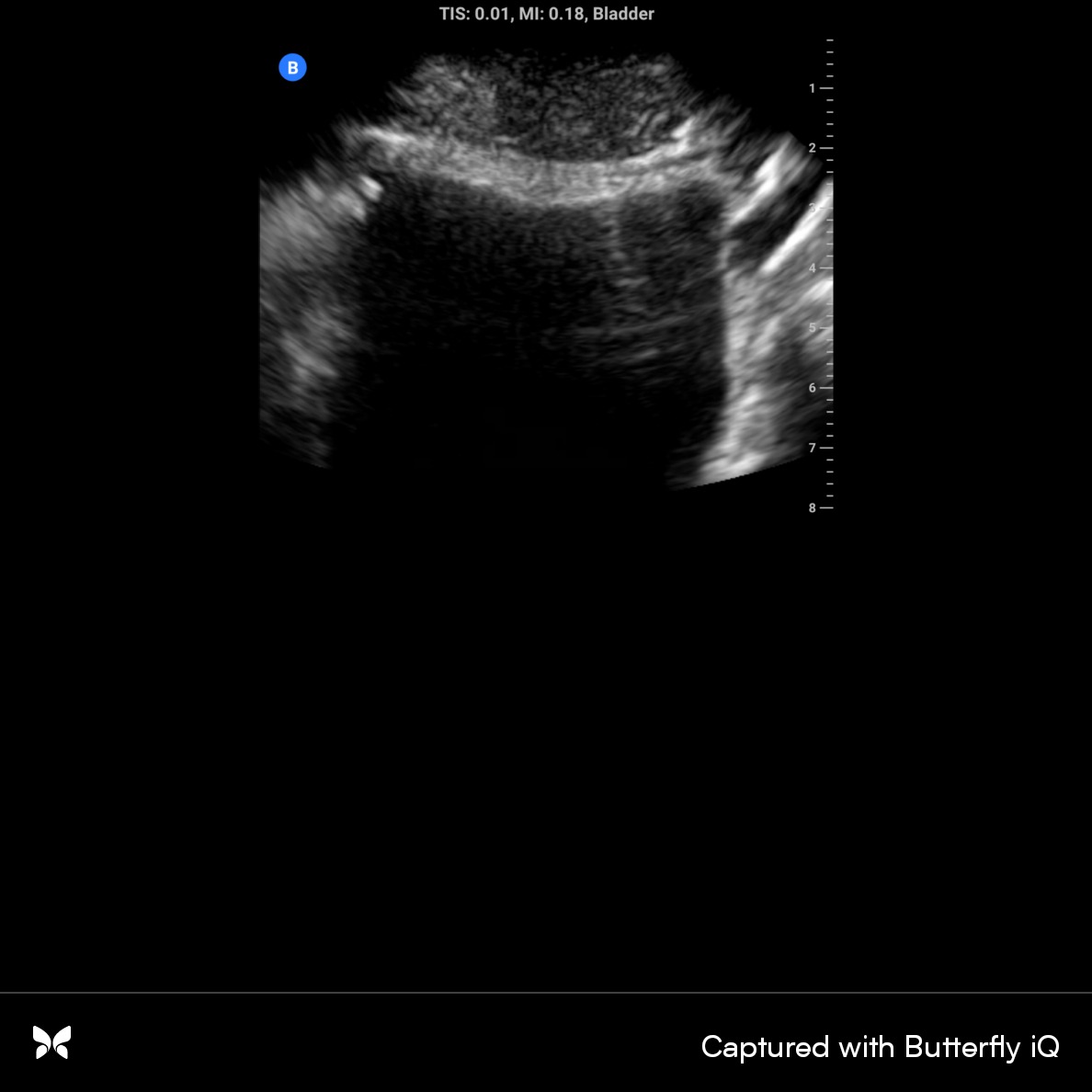


Description: Normal Bladder (Full)


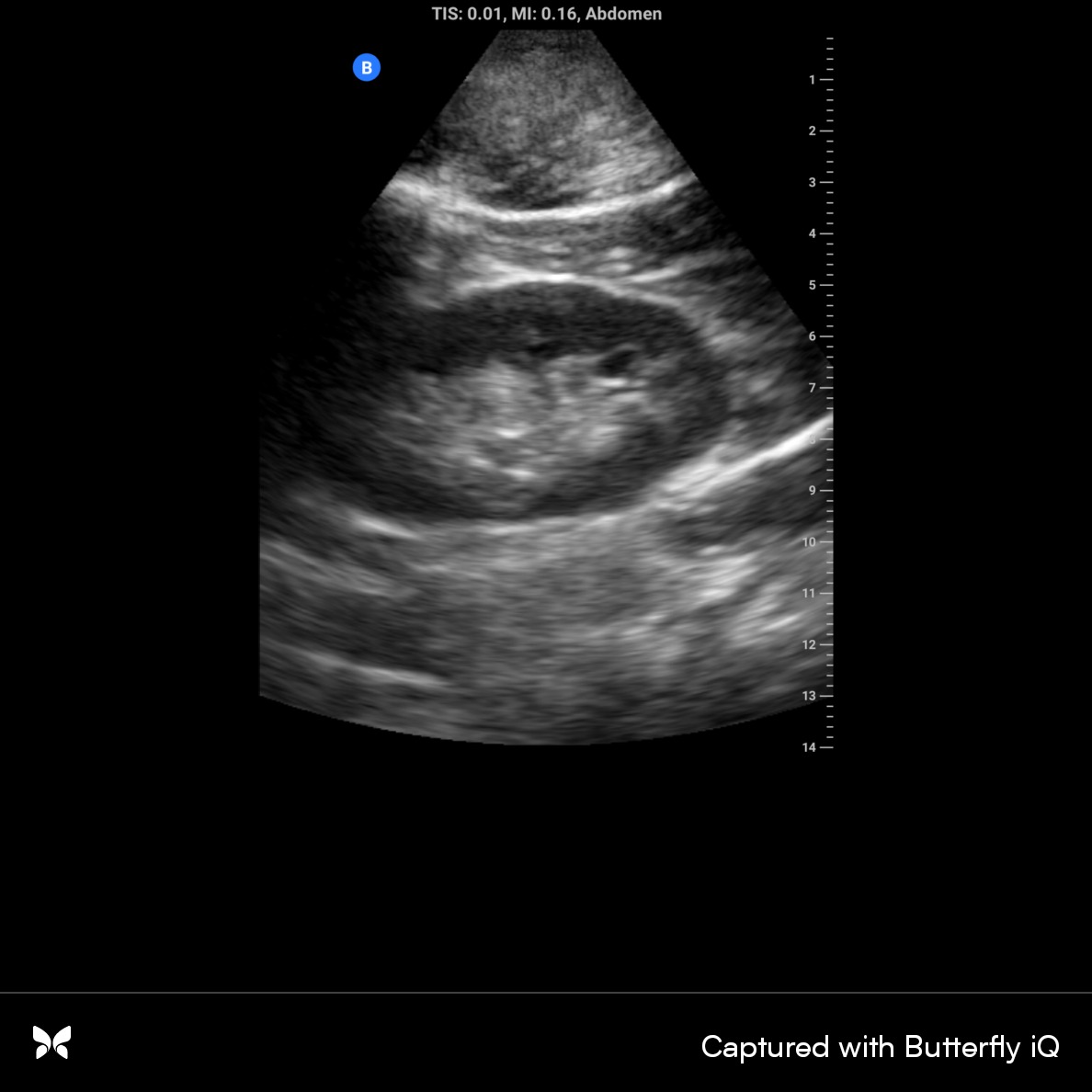


Description: Kidney with cysts


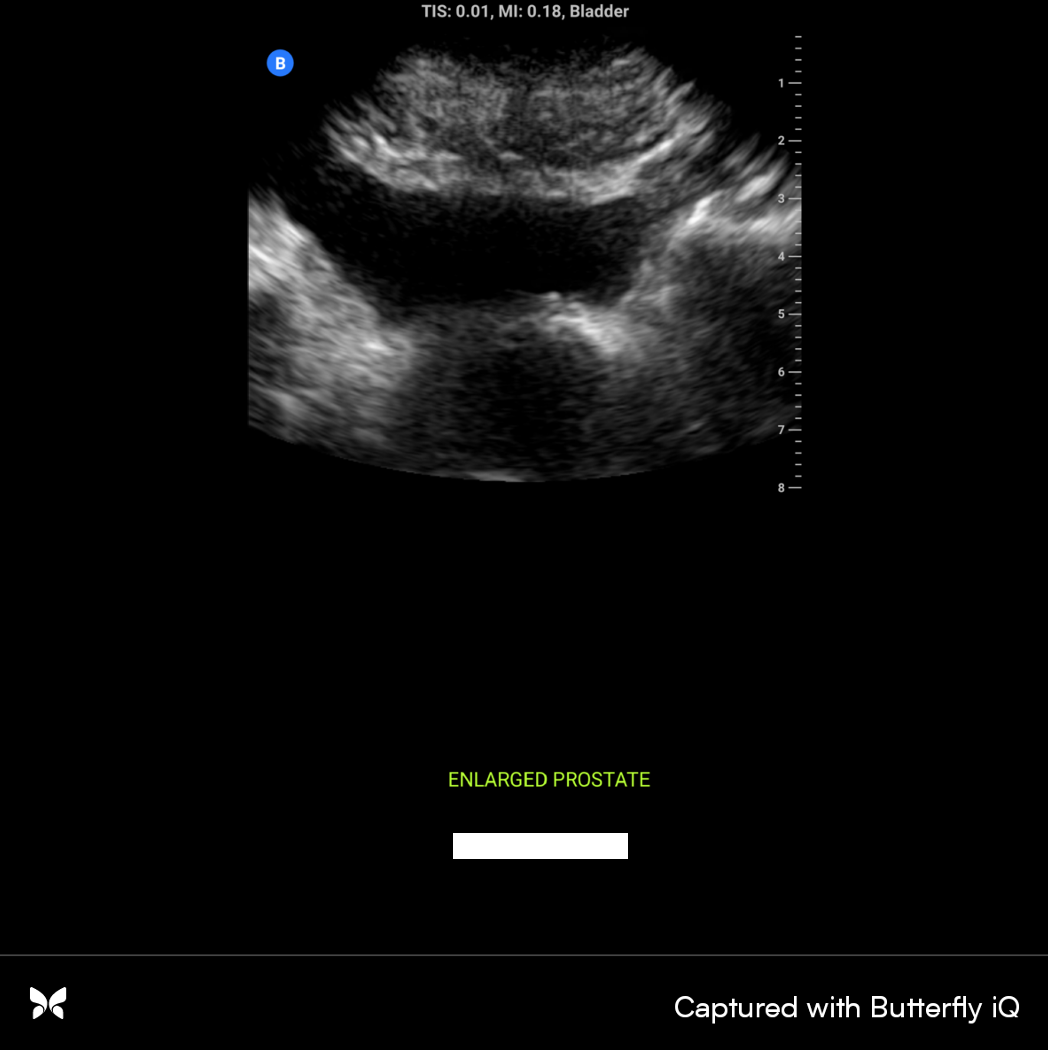


63 y/o male patient presented with difficulty urinating and frequent urination. Ultrasound of the Bladder (axial view) shows an enlarged hyperechoic Prostate gland that decreases the diameter of the Bladder. Mass lesions were absent in bladder. Findings consistent with Benign Prostatic Hyperplasia.


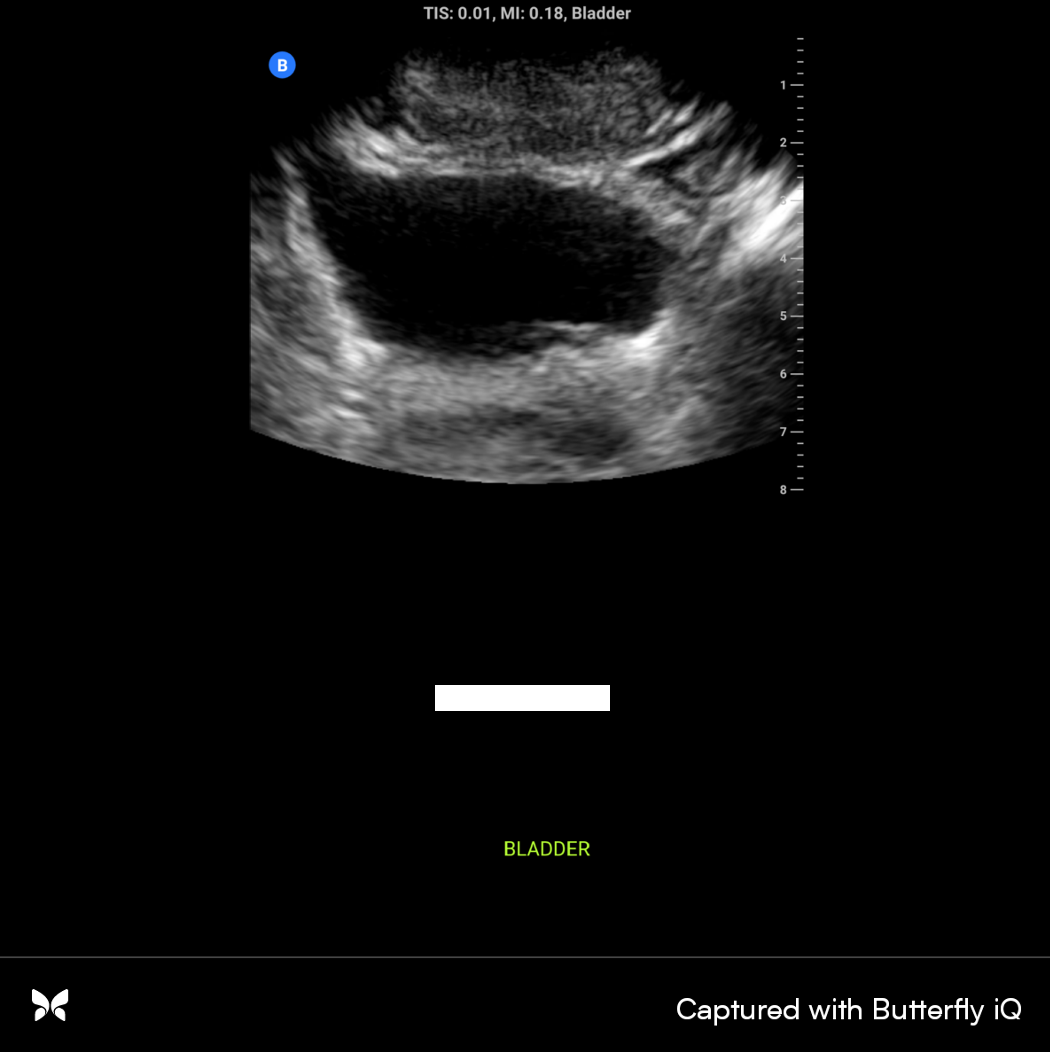


30 y/o male patient presented with left abdominal flank pain, that radiated to groin. Ultrasound of the Bladder (axial view) shows anechoic bladder. Mass Lesions were absent in the bladder. Prostate inferior to the bladder. Findings consistent with a normal Bladder


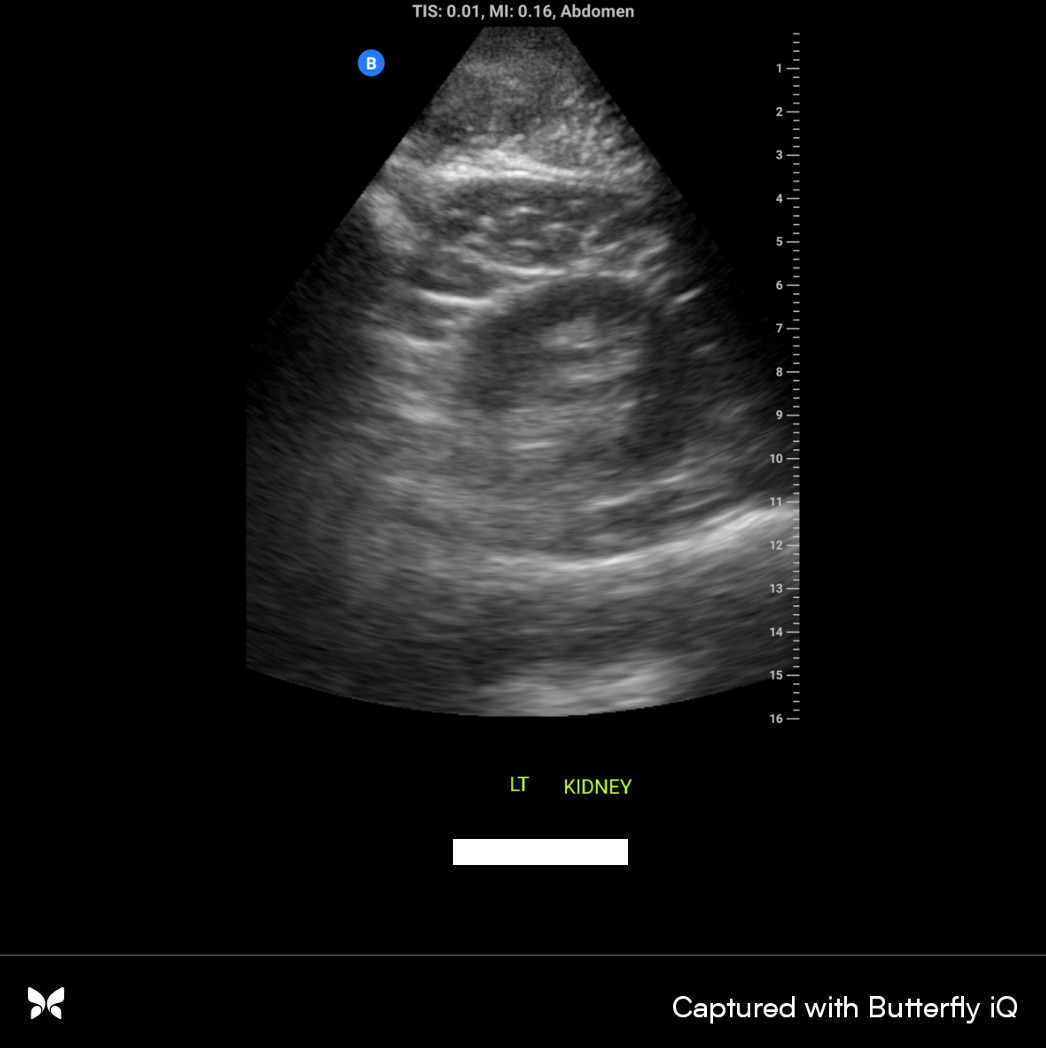


30 y/o male patient presented with left abdominal flank pain, that radiated to groin. Ultrasound of the Left Kidney (longitudinal view) shows no dilation of the renal pelvis or calyces. Mass lesion (caliculi) were absent. Findings are consistent with a normal Left Kidney


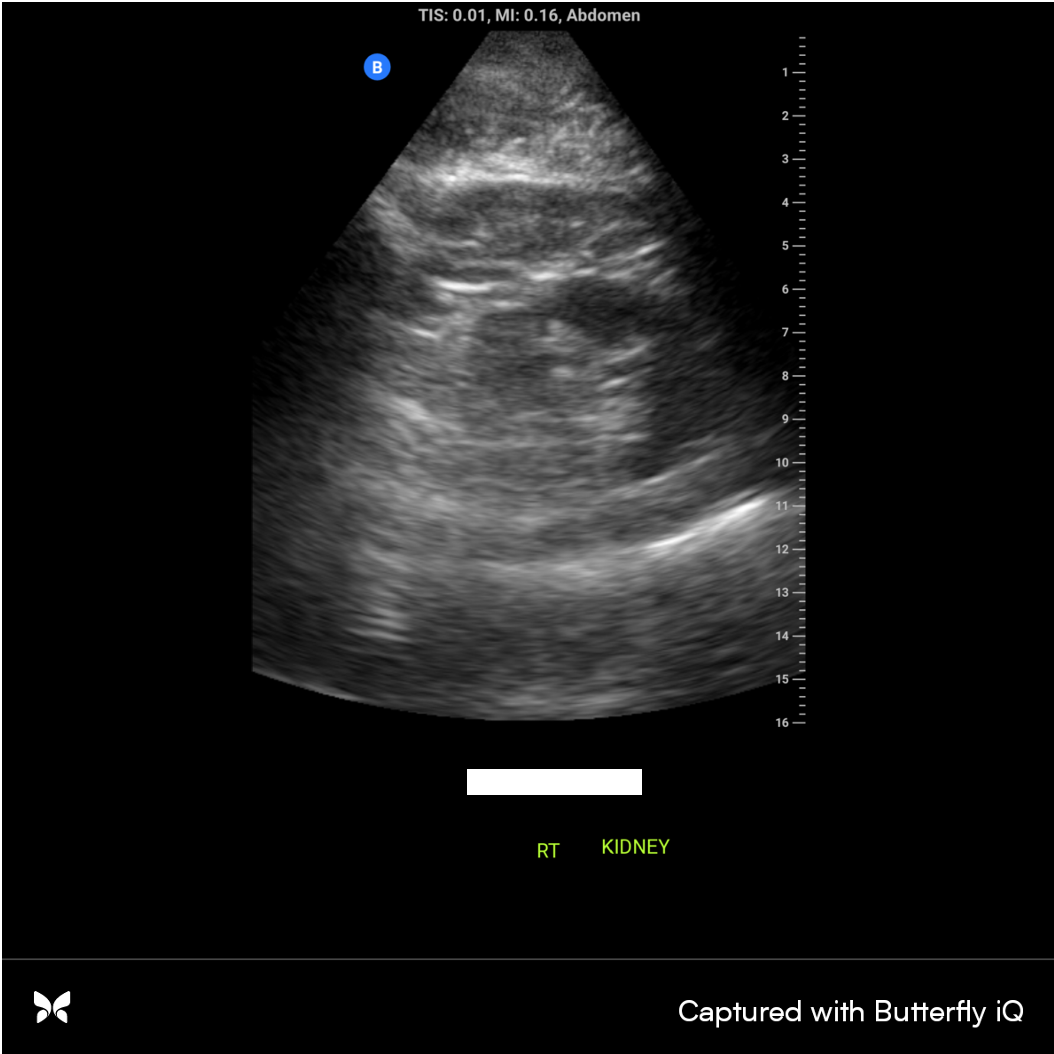


30 y/o male patient presented with left abdominal flank pain, that radiated to groin. Ultrasound of the Right Kidney (longitudinal view) shows no dilation of the renal pelvis or calyces. Mass lesion (caliculi) were absent. Findings are consistent with a normal Right Kidney.


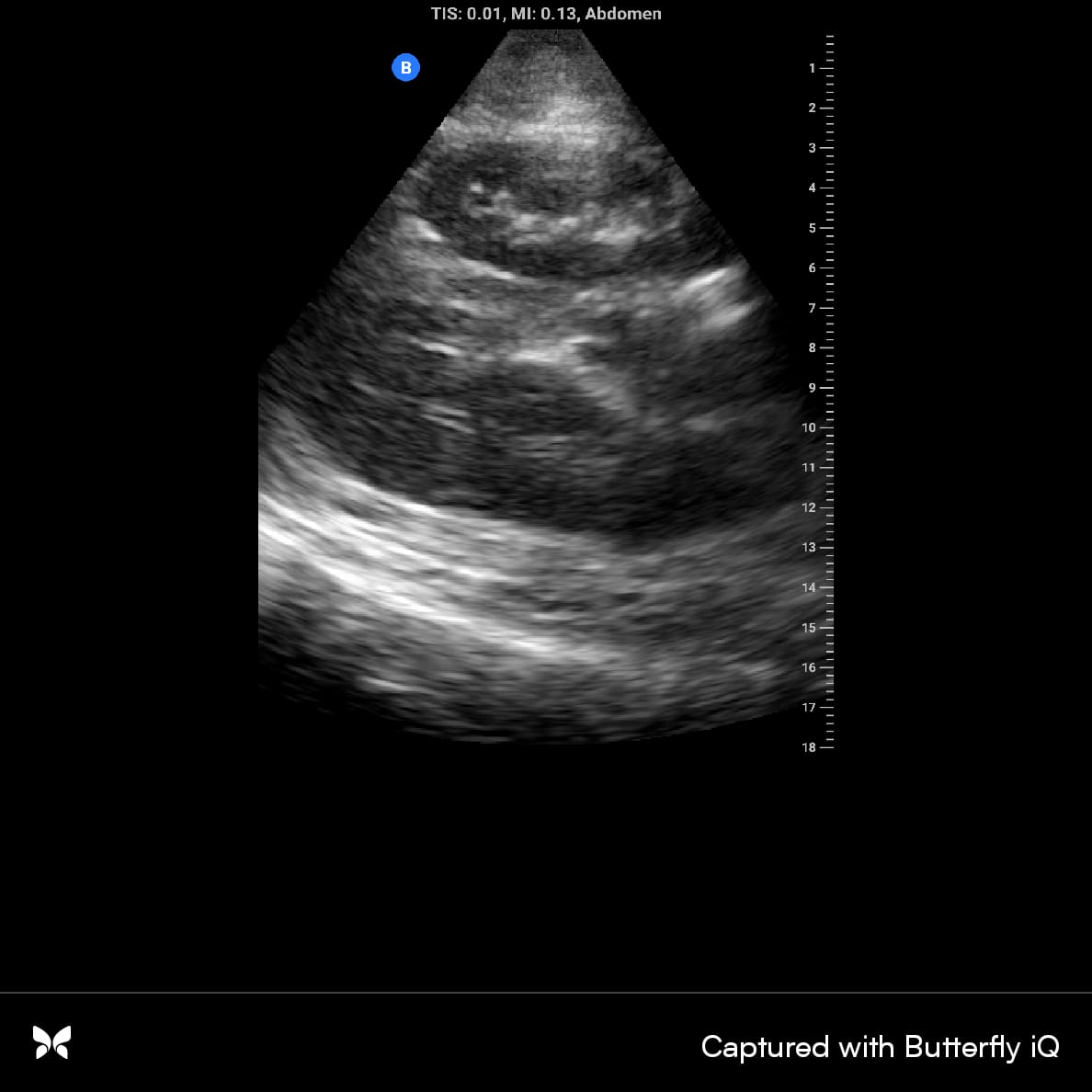


23 y/o female pt with no underlying health conditions. An ultrasound of the kidney in longitudinal view was performed. Upon examination, shows no dilation of the renal pelvis or calyces. No masses appreciated and no renal caculi was observed

Conclusion: the findings were unremarkable


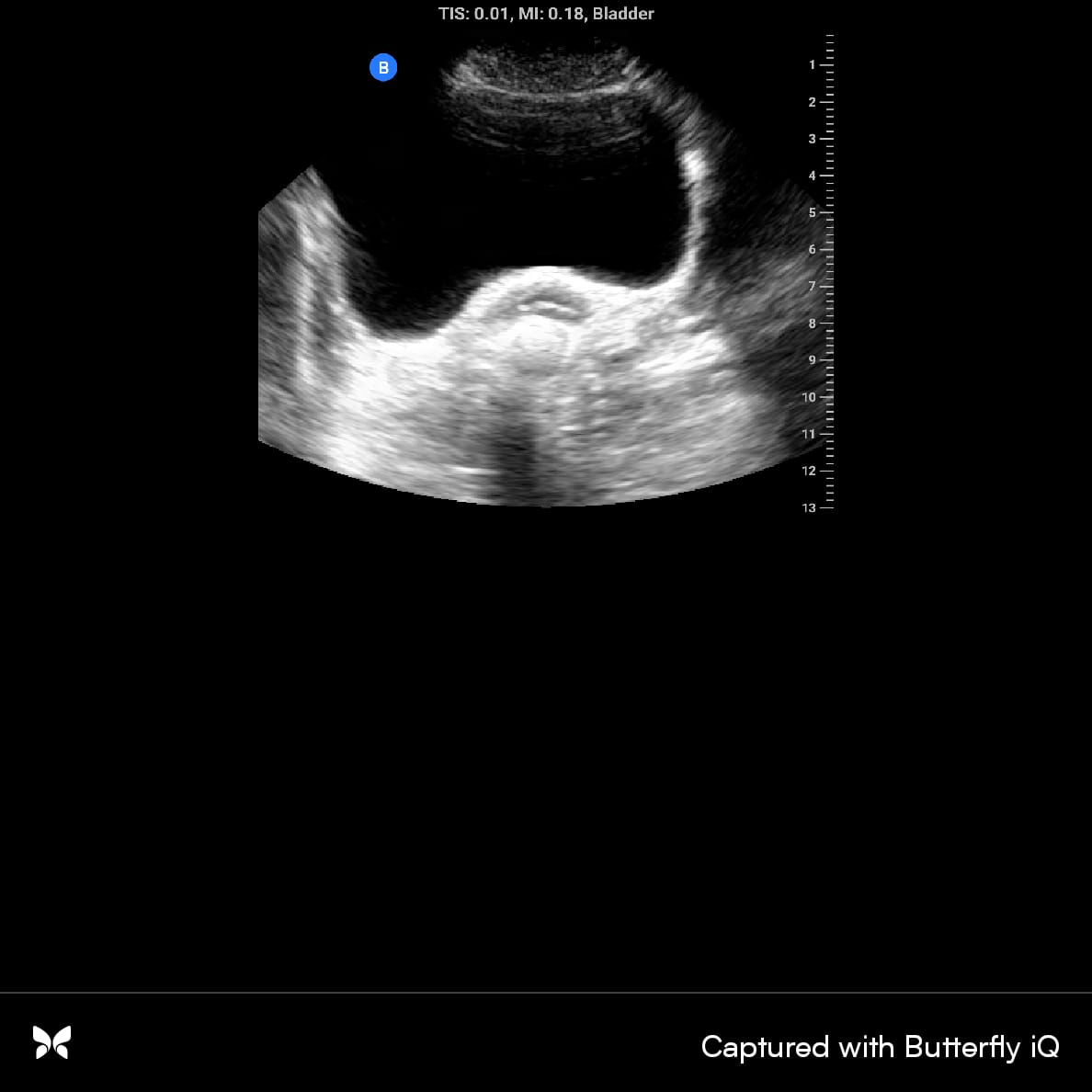


23 y/o female pt with no underlying health conditions. An ultrasound of the bladder was performed under the axial view. Upon examination, wall thickness appears normal. Shows no calculi, outpouchings or obvious masses

Conclusion: findings were unremarkable


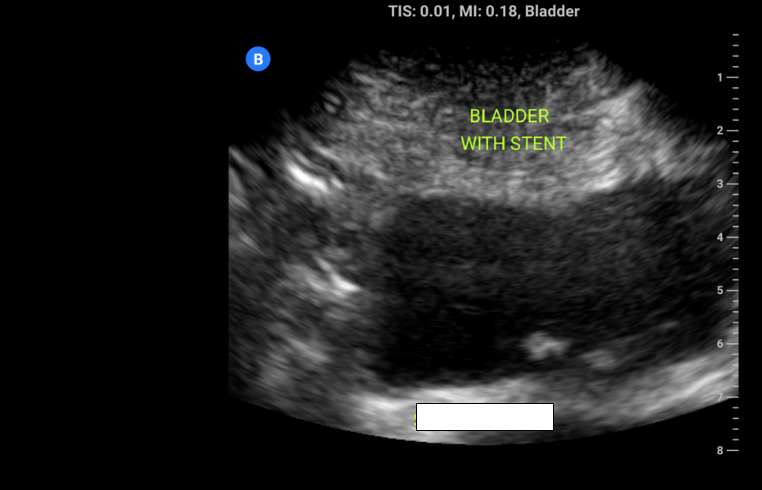


40-year-old male with a past medical history of Nephrolithiasis. Axial view of bladder shows anechoic bladder with urine and hyperechoic stent in the inferior aspect.


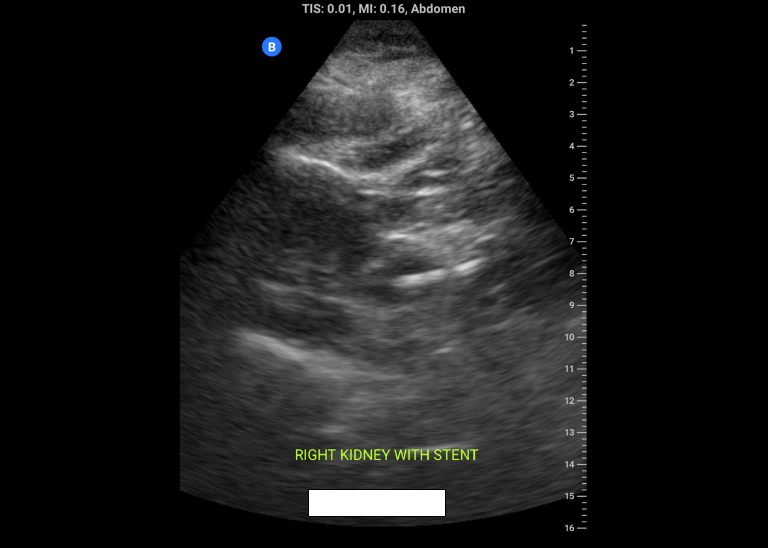


40-year-old male with past medical history of Nephrolithiasis. Longitudinal view of right kidney reveals hyperechoic stent. No renal calculi or dilatation renal pelvis or calyces were appreciated.


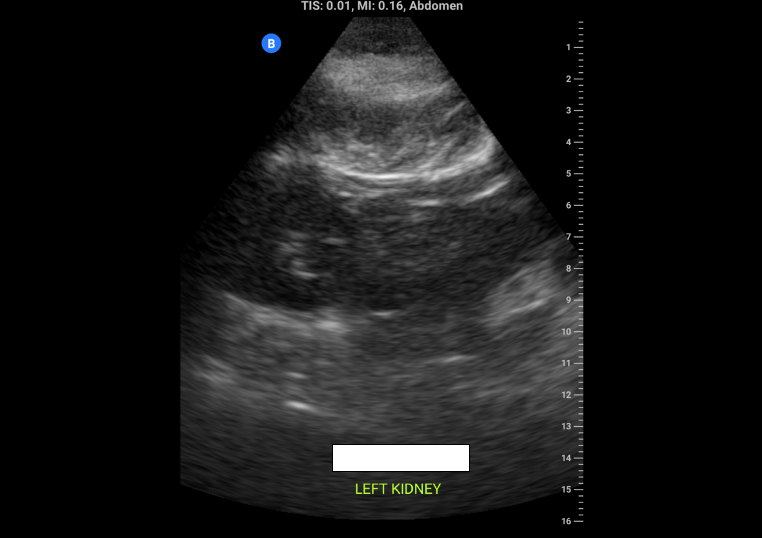


40-year-old male with past medical history of Nephrolithiasis. Longitudinal view of left kidney reveals was normal. No mass lesions or perinephric fluid collection were appreciated.
