# Supplementary material for: Training and implementation of handheld ultrasound technology at Georgetown Public Hospital Corporation in Guyana: a virtual learning cohort study
Source: J Educ Eval Health Prof. 2023 Apr 4;20:11. doi: 10.3352/jeehp.2023.20.11 (PMC11009011; doi:10.3352/jeehp.2023.20.11)
Supplement: Supplementary file 6 — Supplement 1. Written exam. [file jeehp-20-11-suppl1.docx]

**Supplement 1.** Written exam

| Q1. Which probe is best for transabdominal scanning and why is this particular probe the most suitable? |
| --- |
| Q2. What tissues appear white and what tissues appear black on an ultrasound image? |
| Q3. How do you differentiate urine in the bladder from a peritoneal fluid collection? |
| Q4. What is the Society of Fetal Urology criteria for grade 3 hydronephrosis? |
| Q5. When can catheterized urine volume be a better option than bladder ultrasound volume measurement? |
