# Supplementary material for: Training and implementation of handheld ultrasound technology at Georgetown Public Hospital Corporation in Guyana: a virtual learning cohort study
Source: J Educ Eval Health Prof. 2023 Apr 4;20:11. doi: 10.3352/jeehp.2023.20.11 (PMC11009011; doi:10.3352/jeehp.2023.20.11)
Supplement: Supplementary file 7 — Supplement 2. Objective structured clinical exam. [file jeehp-20-11-suppl2.docx]

**Supplement 2.** Objective structured clinical exam

| Within each of the 3 sessions, the learner must: | Point achieved | Comments |
| --- | --- | --- |
| **1. Preparation for the scan**  (a) Greets the patient appropriately and identify the patient with the notes |  |  |
| (b) Confirms that the indication for the procedure is within own competency |  |  |
| (c) Positions the patient correctly |  |  |
| (d) Demonstrates appropriate attitude and professional manner |  |  |
| **2. The scan**  (a) Sets up the equipment acceptably |  |  |
| (b) Probe handling and scanning technique |  |  |
| (c) Identifies the left kidney, right kidney, able to obtain sagittal and axial views |  |  |
| (d) Measures bladder cranial-caudal, anterior-posterior, and right-left planes accurately before and after void |  |  |
| (e) Identifies the prostate if applicable |  |  |
| (f) Able to save or print image |  |  |
| **3. Post-scan**  (a) Informs the patient appropriately |  |  |
| (b) Makes a record of the findings |  |  |
| (c) Interprets and reports findings appropriately |  |  |
| (d) Knows if a repeat scan would be useful |  |  |
